# Deep learning-enabled temporal sequencing of metasurface for rewritable and customizable electromagnetic illusions

**DOI:** 10.1093/nsr/nwag263

**Published:** 2026-05-08

**Authors:** Haoran Han, Jiwei Zhao, Huan Lu, Yongqiang Liu, Bin Zheng, Rongrong Zhu, Hongsheng Chen

**Affiliations:** State Key Laboratory of Extreme Photonics and Instrumentation, ZJU-Hangzhou Global Scientific and Technological Innovation Center, Zhejiang University, Hangzhou 310027, China; The Electromagnetics Academy at Zhejiang University, College of Information Science and Electronic Engineering, Zhejiang University, Hangzhou 310027, China; Zhejiang Key Laboratory of Intelligent Electromagnetic Control and Advanced Electronic Integration, Jinhua Institute of Zhejiang University, Zhejiang University, Jinhua 321099, China; State Key Laboratory of Extreme Photonics and Instrumentation, ZJU-Hangzhou Global Scientific and Technological Innovation Center, Zhejiang University, Hangzhou 310027, China; The Electromagnetics Academy at Zhejiang University, College of Information Science and Electronic Engineering, Zhejiang University, Hangzhou 310027, China; Zhejiang Key Laboratory of Intelligent Electromagnetic Control and Advanced Electronic Integration, Jinhua Institute of Zhejiang University, Zhejiang University, Jinhua 321099, China; State Key Laboratory of Extreme Photonics and Instrumentation, ZJU-Hangzhou Global Scientific and Technological Innovation Center, Zhejiang University, Hangzhou 310027, China; The Electromagnetics Academy at Zhejiang University, College of Information Science and Electronic Engineering, Zhejiang University, Hangzhou 310027, China; Zhejiang Key Laboratory of Intelligent Electromagnetic Control and Advanced Electronic Integration, Jinhua Institute of Zhejiang University, Zhejiang University, Jinhua 321099, China; National Key Laboratory of Scattering and Radiation, Beijing 100854, China; State Key Laboratory of Extreme Photonics and Instrumentation, ZJU-Hangzhou Global Scientific and Technological Innovation Center, Zhejiang University, Hangzhou 310027, China; The Electromagnetics Academy at Zhejiang University, College of Information Science and Electronic Engineering, Zhejiang University, Hangzhou 310027, China; Zhejiang Key Laboratory of Intelligent Electromagnetic Control and Advanced Electronic Integration, Jinhua Institute of Zhejiang University, Zhejiang University, Jinhua 321099, China; School of Information and Electrical Engineering, Hangzhou City University, Zhejiang 310015, China; State Key Laboratory of Extreme Photonics and Instrumentation, ZJU-Hangzhou Global Scientific and Technological Innovation Center, Zhejiang University, Hangzhou 310027, China; The Electromagnetics Academy at Zhejiang University, College of Information Science and Electronic Engineering, Zhejiang University, Hangzhou 310027, China; Zhejiang Key Laboratory of Intelligent Electromagnetic Control and Advanced Electronic Integration, Jinhua Institute of Zhejiang University, Zhejiang University, Jinhua 321099, China

**Keywords:** reconfigurable intelligent metasurface, time-division modulation, task-driven design, electromagnetic illusion, synthetic aperture imaging

## Abstract

The realization of rewritable and customizable electromagnetic illusions fundamentally hinges upon the ability to achieve precise spatiotemporal control over electromagnetic waves. Conventional metasurfaces are confined to globally stationary, periodic protocols, lacking the information entropy to orchestrate complex illusion patterns. Here, we introduce a modular metasurface time-domain programming framework that organizes discrete temporal modulation waveforms as reusable modulation units within a predefined library. By flexibly selecting and sequencing these units, rather than redesigning the entire control law for each new task, the framework redistributes the spectral components of the scattered field to synthesize diverse electromagnetic illusions. A deep generative model is established to map target illusions directly to specific time-domain modulation sequences. The metasurface executes these signals across distinct pulses to physically realize the user-defined illusion. Validated on a synthetic aperture imaging testbed, the system rewrites periodic baselines and synthesizes representative aperiodic illusion patterns under user-specified inputs, achieving high fidelity between intended objectives and measurements (structural similarity index ≥0.91). This work establishes a practical route from target-scene specification to executable metasurface control and provides a scalable paradigm for task-driven wave manipulation in radar imaging scenarios.

## Teaser

A deep-learning-enabled time-modulated metasurface translates desired radar illusion patterns into executable modulation sequences for customizable electromagnetic illusion synthesis.

## INTRODUCTION

Customizing electromagnetic scattering according to a desired target scene is a longstanding objective in wave engineering [1–6]. In radar and imaging applications, this objective can be formulated as the generation of a user-defined illusory scene within the observer’s reconstructed field of view. This process requires a method to directly map a specific user intent to the precise electromagnetic wave modulation commands needed to produce it. Metasurfaces have emerged as the essential hardware foundation for this purpose. These planar arrays of subwavelength elements are capable of tailoring amplitude, phase, and polarization, thereby enabling compact and versatile wavefront engineering across domains ranging from microwaves to optics [7–10]. By precisely designing unit-cell responses and their arrangement, metasurfaces have delivered holography, illusion, and invisibility, as well as computational and sensing functionalities [11–16]. Despite these hardware advances, we still lack a capability to automatically convert a desired illusion into the actual physical control signals required to generate it.

The realization of such customizable illusions is fundamentally constrained by prevailing metasurface control paradigms. Static spatial modulation, implemented through fixed patterning, provides high spatial complexity but cannot be altered once fabricated. It is effective but inherently non-adaptive [17,18]. Dynamic metasurfaces incorporate tunable elements to enable reconfigurability, although existing schemes generally allow the generation of only a single feature at a time, even if its position can be adjusted [19,20]. Temporal modulation offers a pathway to higher complexity by switching metasurface states over time [21–23]. Unlike static or tunable approaches, a single hardware platform can produce multiple distinct responses through temporal sequencing to enable substantially richer electromagnetic behaviors. However, most temporal schemes are low-dimensional and periodic, relying on single-harmonic or simple repetitive waveforms [24–29]. These systems enforce a time-invariant control law in which a fixed modulation cycle repeats indefinitely. Consequently, the expressible patterns tend to be predictable and grid-like, falling short of the arbitrary, aperiodic illusions demanded by complex or deceptive scenarios [30–36]. These regular modulation strategies do not provide sufficient control freedom to synthesize arbitrary user-defined illusion patterns, especially when the desired scene is spatially complex. A critical gap therefore persists regarding how to bridge the difference between a desired visual objective and the aperiodic modulation instructions required for its faithful physical realization.

To surmount these limitations, we introduce a modular temporal programming framework. The total interaction duration required for illusion generation is partitioned into a sequence of independent and reconfigurable time segments [37,38]. In biological systems, different selections and combinations of basic genes form different genotypes, which in turn determine distinct phenotypes, such as differences in appearance, morphology, and other observable biological traits [39,40]. Inspired by this modular and recombinable logic, the temporal modulation waveform applied to the metasurface within each discrete time segment can be viewed as a basic modulation unit. By selecting these units from a predefined modulation-waveform library and arranging them across the time segments involved in illusion generation, the metasurface can produce different illusion patterns. Compared with conventional global coding strategies, this modular scheme avoids repeated redesign of the entire temporal control law for each new task, because the required response can be constructed through flexible selection and recombination from a predesigned waveform library. In this way, different modulation waveforms can be assigned to different probing pulses, and their collective action redistributes electromagnetic features across multiple time scales, thereby enabling the synthesis of diverse and rewritable electromagnetic illusions.

Recent advances in the integration of programmable metasurfaces with neural-network-enabled inverse design have made user-customizable electromagnetic illusion generation feasible [41–47]. To operationalize this capability, we develop a vision-to- sequence compiler powered by a deep neural network. This model establishes the mapping relationship between a target illusion and the corresponding metasurface temporal modulation sequence. Rather than searching an intractable combinatorial space, the compiler directly predicts the pulse-by-pulse modulation unit required for the task. The resulting modulation sequence is executed in real time on a field-programmable gate array (FPGA) to drive a programmable metasurface, thereby expressing the intended electromagnetic illusion as perceived by the observer. Using a rail-based synthetic aperture radar (SAR) testbed, we experimentally demonstrate the synthesis of user-specified and aperiodic illusory scenes with high fidelity. These results confirm the efficacy of our unified pipeline in establishing a direct compilation pathway that transforms user-defined illusions into executable metasurface control schemes. By using inverse design to translate user-defined illusion requirements directly into executable metasurface modulation sequences, our approach enables the automatic generation of target two-dimensional electromagnetic illusions. This capability provides a foundational blueprint for task-driven intelligent systems, equipping them with the autonomy to perceive and physically sculpt their electromagnetic surroundings for applications in radar imaging and electromagnetic deception.

## RESULTS

**Figure 1.**
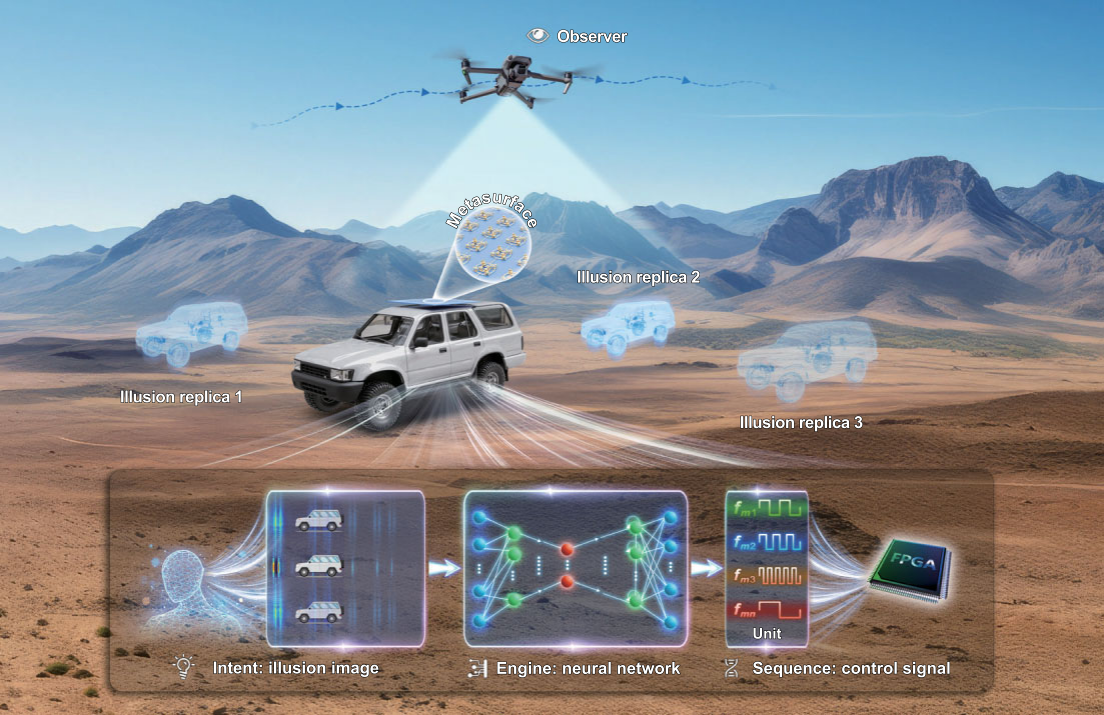
A task-driven paradigm for intelligent customizable illusion synthesis. A user defines a visual intent (the desired illusion). An inverse design network translates the desired illusion into a digital, aperiodic temporal modulation sequence. The time-modulated metasurface sequentially executes these modulation units across distinct time segments, physically sculpting incident waves to render the desired illusion. Finally, following signal accumulation and processing over the imaging duration, the observer perceives the intended illusions, which possess no physical presence, effectively fulfilling the purpose of deception. This marks a paradigm shift from parameter-driven control to holistic, task-driven programming.

We implement the temporal sequencing strategy of the metasurface as a tangible framework synergistically composed of an intelligent compiler and a physical platform (Fig. 1). At its core is a vision-to-sequence intelligent compiler driven by a deep neural network. Within this architecture, discrete temporal modulation waveforms applied to the metasurface at specific moments are viewed as fundamental modulation units, while the complete pulse-wise sequence governing differential temporal modulation over the detection cycle constitutes the executable modulation sequence for illusion generation. The compiler learns the underlying physical mapping between a desired scene and its corresponding modulation sequence. Upon receiving a user-defined illusion image, it generates the precise sequence required to drive the metasurface with differential time-varying modulations. This approach addresses the limitations of conventional globally stationary time modulation, realizing a direct and rapid execution pathway from abstract user intent to physical implementation.

Physically, the predicted modulation sequence is instantiated as a time-varying voltage sequence executed in real time by an FPGA. These signals dynamically bias the tunable elements, thereby switching the reflection states of the metasurface. Governed by the theory of time modulation, these distinct temporal modulation waveforms induce specific spectral redistributions and Doppler shifts within the echo. Leveraging this strategy of time-division modulation, the system precisely expresses the task-specific electromagnetic illusion within the field of view of the observer. As demonstrated in Fig. 1, the lightweight and integrable nature of the system facilitates seamless deployment on high-value platforms such as vehicles. When a user defines a target illusion, the compiler maps this visual objective to executable temporal modulation waveforms, enabling the observer to perceive the intended scene. Notably, such illusions possess no physical presence in real space but materialize exclusively in the observer’s domain, achieving image-domain false-illusion effect.

### Time-division temporal programming strategy for illusion generation

**Figure 2.**
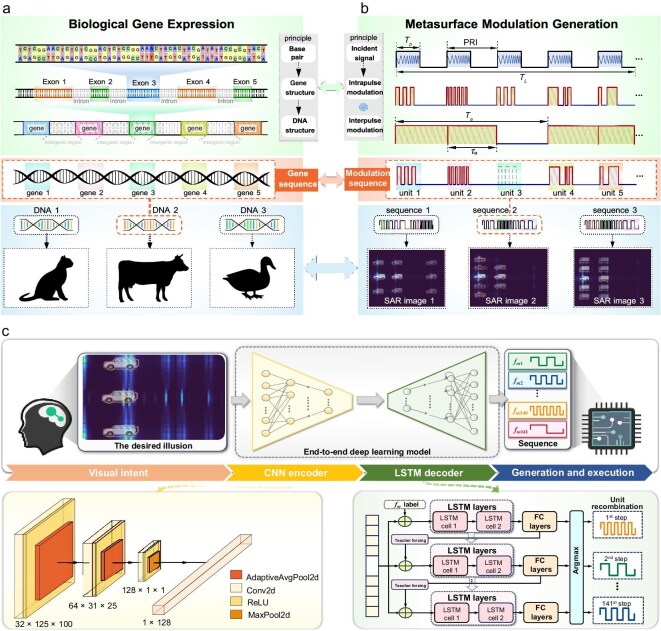
Temporal sequencing strategy and the vision-to-sequence compiler. (a) In biological systems, the genetic information encoded in DNA sequences is processed by intricate gene regulatory networks, which orchestrate the genes into observable traits that underpin phenotypic diversity. (b) Each temporal modulation waveform of the metasurface is analogized as a modulation unit. A comprehensive time-sequenced strategy thus constitutes a synthetic temporal modulation sequence, which fundamentally dictates the spatial and spectral characteristics of the resulting illusion pattern. (c) A deep generative network acts as the vision-to-sequence compiler. It takes a desired illusion image as input, extracts its features with a Convolutional Neural Network encoder, and synthesizes the required temporal modulation sequence with an LSTM decoder.

We employ a biological analogy to facilitate the description of the control variables of the metasurface, motivated by the fact that rich phenotypic diversity in nature arises from the combinatorial organization of basic genetic elements (Fig. 2a) [39,40]. Similarly, diverse electromagnetic illusions can be synthesized through the structured coding of wave control from the metasurface. In the representative imaging scenario illustrated in Fig. 2b, the formation of a single scene relies on the coherent integration of echoes over an observation window ${T}_{\rm L}$ comprising a sequence of ${N}_{\rm L}$ probing pulses. Within this framework, we define the specific temporal modulation waveform of the metasurface applied within each individual pulse duration ${T}_{\rm p}$ as a fundamental modulation unit. In advance, a predefined library comprising a diverse array of temporal modulation waveforms is established to serve as the candidate units. By sequentially selecting distinct units from this library and applying them to the corresponding pulses at different slow time moments, the metasurface imposes highly differentiated temporal modulation across the entire observation timeline. Consequently, the final illusion perceived by the detector arises from the cumulative contribution of these differentially modulated pulses. Crucially, the diversity of achievable illusions is governed by the temporal ordering and the combinatorial arrangement of the modulation units. This mapping between the inter-pulse temporal modulation sequence and the generated complex electromagnetic illusion constitutes the core physical mechanism of the proposed framework.

At the fundamental physical level, the temporal modulation waveform within a single pulse serves as the basic mechanism for intra-pulse spectral control. The time-domain reflection coefficient of the metasurface $\Gamma ( {\hat{t}} )$ is be viewed as a functional unit, while technically it corresponds to the minimal temporal modulation unit applied within one pulse interval. To facilitate a rigorous analytical description, we model the modulation pattern within a single probe pulse ${T}_p$ as a periodic function with a period ${T}_m$ that is discretized into *M* equidistant time slots. For each specific slot m, the reflection coefficient maintains a constant complex value denoted by ${\Gamma }_m$. Consequently, this discrete time-domain function yields a Fourier series expansion expressed as:


(1) \begin{eqnarray*}
\Gamma \left( {\hat{t}} \right) = \mathop \sum \limits_{k = - \infty }^\infty {c}_k{\mathrm{exp}}\left( {j2\pi k{f}_m \hat{t}} \right),
\end{eqnarray*}

where ${f}_m$ represents the modulation frequency of the metasurface. Crucially, the Fourier coefficient ${c}_k$, which determines the weight of the *k*th harmonic, is determined by the ${\Gamma }_m$ (see Methods). This mathematical derivation confirms that distinct temporal modulation units can induce specific and differentiated manipulations of the electromagnetic spectrum. Accordingly, a candidate unit library comprising multiple metasurface temporal modulation waveforms can be constructed for subsequent dynamic selection.

Extending to the system level, the pulse-wise control sequence governs inter-pulse spectral diversity. We analyze this operational mechanism within a synthetic aperture imaging framework characterized by distinct fast and slow time scales. In this framework, different temporal modulation units are assigned across successive pulses, thereby introducing an additional modulation dimension along the slow-time axis ${t}_m$. For rigorous analytical modeling (see Methods), we define the inter-pulse modulation as a pulse-wise sequencing function $F( {{t}_m} )$ that repeats with a period ${T}_a$. The variation of the metasurface reflection coefficient under this pulse-wise temporal modulation yields the expression:


(2) \begin{eqnarray*}
{\Gamma }_{2D}\left( {\hat{t},{t}_m} \right) = \Gamma \left( {\hat{t}} \right) \cdot \left( {\mathop \sum \limits_{q = - \infty }^{ + \infty } {S}_q{\mathrm{exp}}\left( {j2\pi q{f}_a{t}_m} \right)} \right).
\end{eqnarray*}

After range (fast-time) matched filtering, the single-pulse echo can be expressed in the fast time $\hat{t}$ and slow time ${t}_m$ as:


(3) \begin{eqnarray*}
{I}_r\left( {\hat{t},{t}_m} \right) = \mathop \sum \limits_{k = - \infty }^{ + \infty } {A}_k{T}_p\left( {1 - \left| {\frac{{\hat{t}}}{{{T}_p}}} \right|} \right) {\mathrm{exp}}\left[ {{\varphi }_r\left( {{t}_m} \right)} \right] {\mathrm{sinc}} \Bigg[ {K}_r{T}_p\left( {1 - \left| {\frac{{\hat{t}}}{{{T}_p}}} \right|} \right) \left( {\hat{t} - \frac{{2{R}_0\left( {{t}_m} \right)}}{c} + \frac{{k{f}_m}}{{{K}_r}}} \right) \Bigg],\\
\end{eqnarray*}

where ${\mathrm{sinc}}( x ) = \sin ( {{\mathrm{\pi }}x} )/( {{\mathrm{\pi }}x} )$. After performing two-dimensional matched filtering on the LFM echoes, the resulting imaging response is derived as (see Note S5 for detailed derivation):


(4) \begin{eqnarray*}
{I}_a = \mathop \sum \limits_{q = - \infty }^{ + \infty } \mathop \sum \limits_{k = - \infty }^{ + \infty } {D}_{qk}{\mathrm{sinc}}\Bigg[ {K}_r{T}_p\left( {1 - \left| {\frac{{\hat{t}}}{{{T}_p}}} \right|} \right) \left( {\hat{t} - \frac{{2{R}_0\left( {{t}_m} \right)}}{c} + \frac{{k{f}_m}}{{{K}_r}}} \right) \Bigg]{\mathrm{sinc}} \left[ {{K}_\alpha {T}_L\left( {1 - \left| {\frac{{{t}_m}}{{{T}_L}}} \right|} \right)\left( {{t}_m - \frac{{q{f}_a}}{{{K}_a}}} \right)} \right] \exp \left[ {{\varphi }_a\left( {\hat{t},{t}_m} \right)} \right].\!\!\!\!\!\!\!\\
\end{eqnarray*}

This analytical expression reveals that the precise location and intensity of the generated illusions are controlled by the coupling of the intra-pulse harmonic index *k* and the inter-pulse harmonic index *q*. Specifically, the intra-pulse spectral coefficient ${c}_k$ dictates the range displacement $\Delta r\ \propto k{f}_m$, whereas the inter-pulse sequencing coefficient ${S}_q$ determines the azimuth displacement $\Delta a\ \propto q{f}_a$ (see Methods and Note S5). Therefore, instead of specifically designing a globally uniform temporal coding matrix for the entire observation window, we dynamically select modulation units from the existing temporal modulation waveform library for individual pulses. Through these sequential permutations and combinations, diverse and flexibly rewritable electromagnetic illusions are successfully achieved.

### Vision-to-sequence compiler for inverse design

Although the genomic framework provides a structured representation for metasurface temporal modulation, deriving a specific modulation sequence to generate a desired illusion remains a challenging problem. Theoretically, temporal modulation waveforms can encompass a diverse array of waveform parameters, including frequency, shape, and duty cycle, which dictate unique spectral redistribution effects. To simplify the design space while preserving essential modulation capabilities, we restrict the temporal modulation waveforms to square-wave primitives with a fixed duty cycle of 0.5, treating only the modulation frequency ${f}_m$ as a tunable parameter. Considering the physical constraints of radar range resolution and the maximum detection range (detailed in Note S6), the discrete ${f}_m$ are strictly limited from 0 to 90 Hz in 10 Hz increments. This yields a predefined library of 10 distinct temporal modulation waveforms. In our configuration, the number of pulses is ${N}_L = 141$. The corresponding metasurface modulation sequence is represented as $G = [ {{g}_1,{g}_2,\ldots,{g}_{141}} ]$, where each element ${g}_n$ is selected from the predefined library. Even under this simplification, the combinatorial design space is immense, on the order of 10^141^ in our system, rendering conventional approaches like manual tuning or brute-force search completely infeasible, particularly under real-time constraints [48–50].

To overcome this computational bottleneck, we construct a vision-to-sequence intelligent compiler powered by a deep generative neural network that automates the inverse design process. Achieving on-demand customization critically depends on an engine capable of intelligent selection and recombination from this predefined library. As illustrated in Fig. 2c, our compiler transcends simple matching algorithms by employing an encoder-decoder architecture strategically designed to map illusion patterns into metasurface temporal modulation sequences. The encoder employs a hierarchical Convolutional Neural Network architecture configured to perform progressive feature abstraction and compression. This module consists of three cascaded convolutional units, each comprising a convolutional layer, an activation function, and a pooling operation. Specifically, the first layer utilizes 32 kernels of size $3\ \times {\mathrm{\ }}3$ with a stride of 2, coupled with a ReLU activation function and a $2\ \times {\mathrm{\ }}2$ max pooling layer. This initial stage compresses the input distribution image of the illusion, which possesses dimensions of $501\ \times {\mathrm{\ }}401$, into a feature map of $32\ \times {\mathrm{\ }}125\ \times {\mathrm{\ }}100$. The second layer expands this representation to 64 channels and further downsamples it to a dimension of $64\ \times {\mathrm{\ }}63\ \times {\mathrm{\ }}50$. The third and final abstraction layer applies 128 kernels and introduces an adaptive global average pooling layer, ultimately outputting a compact $1\ \times {\mathrm{\ }}128$ latent feature vector. This highly compressed vector encapsulates the global structure information of the illusion image and serves as the fundamental conditional input for the subsequent sequence generation module.

To bridge spatial illusion features with temporal sequencing of the metasurface, the decoder utilizes a recurrent generation module based on Long Short-Term Memory (LSTM) units. In this mechanism, the modulation unit category label from the previous step is mapped into a 64-dimensional dense vector via an embedding layer to circumvent the dimensionality curse associated with one-hot encoding. The embedding is subsequently concatenated with the 128-dimensional global feature vector derived from the encoder, establishing a fusion strategy that ensures the global visual context actively guides the prediction at every time step to maintain generation consistency. The core generation engine consists of a two-layer stacked LSTM network with 256 hidden units operating in batch-first mode. To optimize convergence and mitigate error accumulation, the training process incorporates a teacher forcing strategy with a probability of 0.5 that balances the injection of ground-truth signals for stability against model-generated signals for generalization. At each time step $t \in \{ {1,2,\ldots,141} \}$, the high-dimensional hidden state is projected via a fully connected layer into a 10-dimensional probability distribution ${\mathrm{O}} = [ {{{\mathrm{o}}}_1,{{\mathrm{o}}}_2,\ldots,{{\mathrm{o}}}_{141}} ]\in {\mathbb{R}}^{\textit{batch}\ \times 141 \times 10}$. A subsequent argmax operation identifies the discrete modulation unit index $G = [ {{g}_1,{g}_2,\ldots,{g}_{141}} ]\in {\mathbb{Z}}^{\textit{batch} \times 141}$, which is physically mapped to the corresponding modulation frequency ${f}_{m,t} = 10 \cdot {g}_t \in \{ {0,10,20,\ldots,90} \}$ Hz. This frequency value is utilized to drive the metasurface in a pulse-by-pulse manner, thereby modulating the probing signal at each specific temporal instance to realize the target spectral response.

Consequently, when provided with a user-defined illusion, the intelligent compiler instantaneously predicts the requisite temporal modulation sequence, which is then executed by the square-wave-modulated metasurface to physically synthesize the intended electromagnetic illusion (see Methods and Note S6 for model details).

### Physical implementation of modulation units with a programmable metasurface

**Figure 3.**
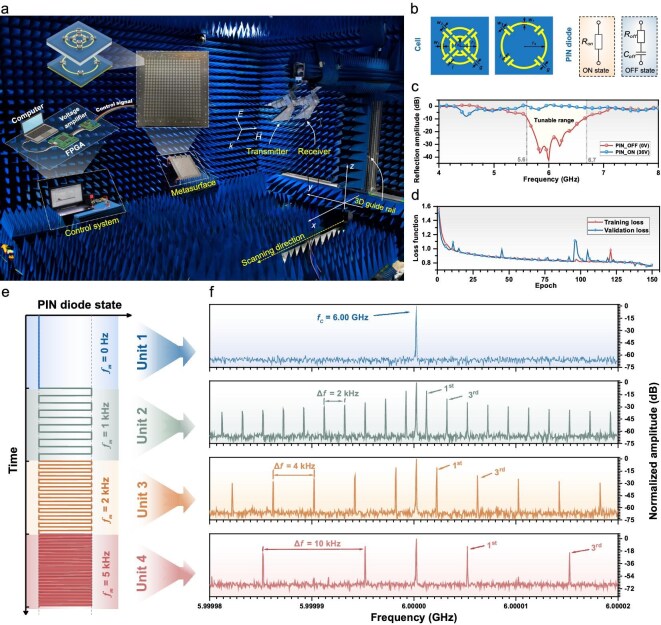
Physical realization of the metasurface compiler. (a) Overview of the rail-based SAR imaging platform, including the programmable metasurface compiler, control system, and transceiver antennas. (b) Schematic of the reconfigurable metasurface cell. A PIN diode is integrated into the metallic structure, enabling the cell to switch between a highly reflective ON state and a highly absorptive OFF state under external voltage control, thereby providing the physical basis for 1-bit modulation. (c) Measured reflection coefficient of the PIN diode in ON and OFF states. (d) Training and validation loss curves of the model. (e) Timing diagram of the modulation units for dynamic control. (f) Measured spectra of the system under the control of different modulation units.

To experimentally validate our framework, we established an integrated hardware-software platform as illustrated in Fig. 3a. This experimental setup features a programmable metasurface positioned at the geometric center of the imaging region to interact with a rail-based SAR system operating within the 5.5–7.5 GHz frequency band. During operation, the system executes a stop-and-go scanning protocol along a 1.4 m rail by transmitting identical frequency-swept signals at ${N}_L=141 $ equidistant spatial positions. The physical execution layer is instantiated on a programmable metasurface comprising a 20 × 20 array of unit cells as detailed in Fig. 3b, which integrate PIN diodes utilizing a column-based series feeding structure (see Note S1). Experimental characterization of the reflection coefficient within a microwave anechoic chamber demonstrates distinct amplitude switching capabilities governed by external voltage control. The measured scattering parameters presented in Fig. 3c reveal that the metasurface exhibits an absorptive response with a reflection magnitude below −10 dB when biased in the OFF state at 0 V. Conversely, the device transitions to a high-reflectance mode upon the application of a 36 V bias for the ON state. This binary response confirms that a square-wave control signal alternating between these two voltage levels effectively modulates the reflection coefficient of the metasurface in real time.

The compiler is implemented using a deep learning model designed to translate user-defined illusion into executable metasurface modulation unit sequences. Utilizing the simplified forward model in Equation (4), we rapidly synthesize a large-scale dataset comprising 77 500 pairs of metasurface temporal modulation sequences and corresponding illusion images. To ensure robust network performance, we extract the distributions of these illusions and applied explicit data augmentations, including random Gaussian noise, amplitude scaling, and retained practical defocusing or energy smearing effects (as detailed in Note S7). The optimization process, governed by a time-step-averaged cross-entropy loss function, demonstrates stable optimization of the training objective without overfitting, as evidenced by the convergence characteristics in Fig. 3d. Quantitative evaluation further supports the effectiveness of the compiler. While the predicted temporal modulation sequences do not always exactly match the preset reference sequences, the synthesized illusion patterns remain in high agreement with the target templates in simulation, as indicated by similarity values exceed 0.9. These empirical results demonstrate that minor isolated deviations in the predicted modulation unit sequence exert a negligible impact on the final imaging outcome and thereby ensure the operational stability of the system (see Notes S7 and S8).

The validated digital modulation sequence is subsequently instantiated within the physical metasurface system. As illustrated in Fig. 3e, an FPGA orchestrates the binary amplitude modulation of the metasurface by translating the abstract sequence into physical voltage waveforms. In this execution stage, each modulation unit corresponds to a square-wave temporal modulation waveform characterized by a distinct modulation frequency that is applied to the metasurface during its designated time segment. To verify the spectral engineering capability of these modulation units, we actively manipulated the spectrum of a single-tone continuous-wave source operating at a carrier frequency of ${f}_c = $ 6 GHz using a spectrum analyzer for recording. As illustrated in Fig. 3e and f, the metasurface is initially driven by Unit 1 corresponding to a static state with ${f}_m = $ 0 Hz, resulting in a measured echo spectrum that remains centered at the carrier frequency. In subsequent time slots, the system sequentially activates Unit 2, Unit 3, and Unit 4, which correspond to square-wave modulation frequencies of ${f}_m = $ 1, 2, and 5 kHz, respectively. Governed by the theory of time-modulated metasurfaces (see Note S2), the backscattered frequency components for a square-wave modulation emerge at $f = {f}_c \pm k{f}_m$ where k represents an odd integer. Consequently, the measured reflection spectra presented in Fig. 3f exhibit precise harmonic distributions wherein the spacing between adjacent odd orders equals $2{f}_m$, corresponding to 2, 4, and 10 kHz for the applied modulation frequencies. These observations confirm that by applying specific square-wave modulation units with distinct frequencies, the system effectively redistributes the electromagnetic energy from the carrier to designated spectral bands. Moreover, since the maximum modulation frequency is constrained by the switching speed of the PIN diodes, these measurements also verify the stable operation of the PIN-diode-based metasurface within the modulation range adopted in this work. The rigorous spectral validation of this integrated platform confirms the precise physical execution of temporal modulation sequences, thereby establishing the supporting evidence for subsequent imaging experiments.

### Experimental demonstration of customizable illusion synthesis

Experimental validation of the proposed framework for programmable illusion synthesis was performed in a stepwise manner, progressing from one-dimensional range profiling to two-dimensional synthetic aperture imaging. To verify the fundamental relationship between temporal modulation sequences and the resulting illusion patterns in one dimension, we conducted range-profile measurements using a transceiver based on a vector network analyzer (VNA) with separate transmitting and receiving horn antennas. The system executed a frequency sweep spanning 5.5–7.5 GHz over a total sweep duration of ${T}_p$ = 1002 ms to yield a 2 GHz bandwidth and the corresponding fine range resolution.

**Figure 4.**
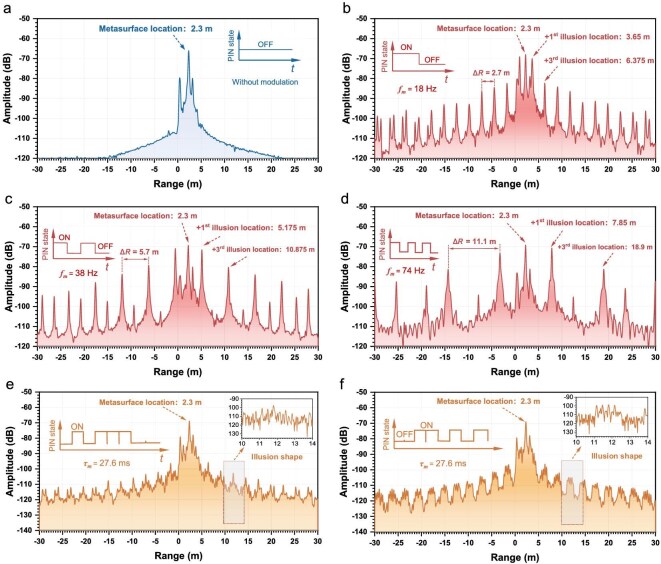
Experimental validation of 1D range-profile illusion generation using different modulation units. (a) Measured one-dimensional range profile of a static metasurface, showing a single reflection peak at its physical location (2.3 m). (b–d) Range profiles under square-wave modulation at 18, 38, and 74 Hz (treated as three independent modulation units), respectively, showing equally spaced odd-order illusion replicas. (e, f) Range profiles generated by two different custom-coded and complex modulation units, demonstrating the creation of illusions with varied spatial positions and unique morphological features.

With the metasurface maintained in a static reflective state at a distance of 2.3 m from the transceiver, the resulting range profile presented in Fig. 4a exhibits a solitary sharp peak corresponding to the actual physical location. Square-wave control signals with distinct modulation frequencies of ${f}_m$ = 18, 38, and 74 Hz are subsequently employed as fundamental modulation units. As shown in Fig. 4b–d, driven by these respective modulation waveforms, the metasurface generates a series of equally spaced illusion replicas in the measured range profiles, establishing a striking contrast with the static baseline shown in Fig. 4a. The measured spatial intervals between adjacent odd-order illusion replicas yielded values of $\Delta R$ = 2.7, 5.7, and 11.1 m. These experimental results agree well with the theoretical predictions of 2.705, 5.711, and 11.122 m, maintaining a relative error consistently below 0.2%. As illustrated in Fig. 4e and f, two distinct voltage modulation waveforms, each defined by a periodicity of M = 8 and a pulse width of ${\tau }_m$ = 27.6 ms, were individually further implemented on the metasurface. This operation resulted in the synthesis of illusion replicas manifesting differentiated morphological contours and cluster-like spatial distributions. A comparative analysis clearly reveals that the resultant electromagnetic illusions are intrinsically governed by the spectral and temporal attributes of the applied temporal modulation waveforms, with differences in frequency and waveform shape leading to distinct illusion morphologies. For simplicity, we establish the modulation unit library using multiple square-wave temporal modulation waveforms with distinct frequencies. To synthesize more diverse illusions, additional candidate units can be referenced in Note S11, or alternative temporal coding metasurfaces can be deployed.

**Figure 5.**
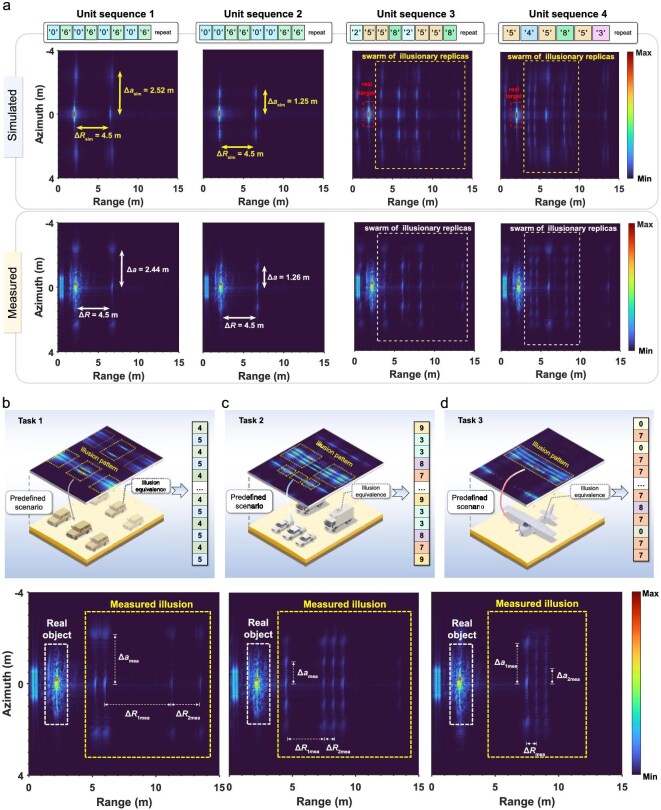
Task-driven synthesis realizes complex and bespoke illusion patterns. (a) A periodic, rule-based temporal modulation sequence emulating classical ISRJ. The experimental result (bottom) matches the simulated grid-like pattern (top), highlighting the limitations of periodic control. (b–d) Experimental realization of task-driven synthesis. For each case, the user-defined visual objective (top) is compared to the experimental measurement (bottom). The system faithfully generates (b) a sparse rectangular array, (c) a dense linear cluster, and (d) a complex heterogeneous pattern, demonstrating the synthesis of arbitrary patterns inaccessible to conventional methods.

Following the validation of the framework in the 1D domain, the approach was extended to the 2D scenario, extending the framework beyond periodic control baselines. For the construction of the combinatorial test set, the modulation-frequency range of the square-wave voltage is spanned from 0 to 90 Hz which is encoded as a discrete modulation unit element, indexed from 0 to 9. As a benchmark, realizing classical intermittent sampling repeater jamming (ISRJ) necessitates capturing the probing signal and intermittently retransmitting it, a complex process that relies heavily on sophisticated signal acquisition and processing chains. In stark contrast, our framework enables the straightforward realization of ISRJ through direct metasurface modulation by programming the two distinct temporal modulation sequences ${G}_1 = $ [0,6,0,6,…,6] and ${G}_2 = $ [0,0,6,6,…,6], as illustrated in Fig. 5a. Under the control of ${G}_1$, the illusionary replicas were arranged in a regular grid with spacing intervals of Δ*R* = 4.5 m in range and Δ*a* = 2.44 m in azimuth. The measured results deviated minimally from simulation results, maintaining a relative error below 4%. Furthermore, two complex temporal modulation sequences ${G}_3 = $ [2,5,5,8,…,2,5,5,8] and ${G}_4 = $ [5,4,5,8,5,3,…,5,3,5] were generated. These sequences utilize active modulation unit elements to replace the traditional intermittent off state (unit 0), thereby rewriting significantly more intricate two-dimensional illusions, with an average SSIM exceeding 94% between simulation and experiment.

Finally, the representative capability of the vision-to-sequence compiler was demonstrated through the synthesis of arbitrary and complex electromagnetic illusion patterns. Three diverse and complex visual objectives were provided: a sparse rectangular array, a dense linear cluster, and a heterogeneous pattern with varying intensities. For each objective, the compiler predicted the required temporal modulation sequence. Subsequently, the metasurface was sequentially modulated in the synthetic aperture imaging measurement setup to execute the generated modulation sequence, resulting in the realization of the intended visual illusion objectives, as visually demonstrated in Fig. 5b–d. To rigorously quantify the robust fidelity visually demonstrated by the synthesized electromagnetic illusions and comprehensively assess the precision of the system, the SSIM was employed via a dual assessment strategy. First, the sequence generation accuracy was evaluated by comparing the predefined illusions with the simulated results driven by the generated modulation sequences. Second, the SSIM values between simulation and experiment were consistently high, measuring 0.9435, 0.9387, and 0.9484 (see Notes S8 and S10). Meanwhile, although the measured results are affected by antenna coupling and residual peaks from the metasurface, the SSIM values between the experimental results and the predefined templates still achieve 0.9558, 0.9153, and 0.9100. Beyond visual assessment, objective physics-based metrics are introduced to rigorously evaluate the illusion generation (provided in Note S12). Specifically, the experimental results demonstrate precise command over spatial sparsity and location (Fig. 5b), the generation of dense, clustered features emulating complex scattering targets (Fig. 5c), and the orchestration of heterogeneous patterns with varying intensities (Fig. 5d). Quantitatively, the relative spatial error for all three illusion generation tasks remains within 5%. Despite the inherent physical constraints of the 1-bit amplitude coding metasurface, which lead to a residual peak at the metasurface location, the system achieves an average energy transfer efficiency of 73.81%. This performance closely approaches the theoretical maximum limit for such architectures. Furthermore, we evaluated the contrast between the synthesized illusions and the ambient environment by calculating the signal-to-clutter ratios. For the tasks illustrated in Fig. 5b, c, and d, the ratios for the strongest illusions are 45.6, 43.6, and 45.8 dB, respectively, while the ratios for the weakest illusions are 27.8, 32.5, and 31.3 dB. This comprehensive validation underscores the capacity of the framework to synthesize representative aperiodic illusion patterns specified by the user, thereby exceeding the inherent limits of conventional manual or periodic rule-based programming approaches for metasurfaces. As summarized in Table 1, the proposed framework advances previous works in three key aspects, including illusion dimensionality, modulation flexibility, and the transition from manual forward design to intelligent inverse design.

**Table 1.** Comparative analysis with existing approaches.

	Coding	Modulation				
Ref.	type	domain	Modulation scheme	Illusion Dimension	Design paradigm	Application scenario
[28]	2-bit	Fast-time only	Global single waveform modulation	1D HRRP	Manual forward	False targets in 1D range profiles
[29]	1-bit	Fast-time only	Global single waveform modulation	1D HRRP	Manual forward	False targets in 1D range profiles
[32]	2-bit	Fast-time and slow-time	Predefined coding matrix	Range–velocity	Manual forward	Disrupting multi-static localization
[33]	1-bit	Fast-time and slow-time	Fixed waveform (identical for all pulses)	1D range-domain distribution	Manual forward	1D distributed targets in ISAR images
[35]	Multi-bit	Fast-time and slow-time	Periodic modulation in slow-time	1D azimuth-domain distribution	Manual forward	1D distributed targets in SAR images
**This work**	**1-bit**	**Fast-time and slow-time**	**Differentiated modulation across different pulses**	**2D SAR illusion (range & azimuth)**	**Intelligent inverse**	**Customizable and complex 2D SAR illusion generation**

## CONCLUSION

In summary, this work introduces and validates a temporal sequencing framework as a structured approach for realizing rewritable and customizable electromagnetic illusions. Within this framework, temporal modulation waveforms are organized as modular, selectable, and recombinable functional units. Through the strategic sequencing of these units, the system achieves differential control over both fast-time and slow-time dimensions, thereby enabling the generation of diverse two-dimensional illusion patterns within the present model and experimental platform.

To further enable on-demand customization, we establish a vision-to-sequence intelligent compiler that maps a target illusion to the corresponding modulation sequences, thus automating the inverse design process and directly translating user-defined visual objectives into executable time-varying voltages. By combining the flexibility of modular temporal sequencing with the automated synthesis capability of the intelligent compiler, this work provides both a foundation and an experimental proof of concept for task-driven electromagnetic illusion engineering. Looking forward, richer coding dimensions and more complex temporal waveforms may further expand the accessible space of the modulation units and illusion diversity, while future radar systems with shorter pulse widths and larger bandwidths will place more stringent demands on modulation hardware, particularly in switching speed and temporal resolution. These advances may further broaden the applicability of the proposed framework to adaptive camouflage, dynamic signature manipulation, and reconfigurable coherent imaging systems.

## METHOD

### Theoretical basis of illusion generation

In a SAR system, the final two-dimensional imaging result ${I}_a( {\hat{t},{t}_m} )$ is achieved by matched filtering in the range and azimuth directions. When the echoes are simultaneously modulated by the periodic intra-pulse signal $\Gamma ( {\hat{t}} )$ and inter-pulse signal $F( {{t}_m} )$ generated by the metasurface, a series of illusion replicas will be produced in the imaging result. The range separation ${\mathrm{\Delta }}R$ and azimuth separation ${\mathrm{\Delta }}a$ between illusion replicas of arbitrary orders can be calculated by:


(5) \begin{eqnarray*}
\Delta R = \frac{{c\left| {{k}_1 - {k}_2} \right|{f}_m{T}_p}}{{2B}},
\end{eqnarray*}

(6) \begin{eqnarray*}
\Delta a = \frac{{\big| {{q}_1 - {q}_2} \big|{f}_a}}{{{K}_a}} \cdot v,
\end{eqnarray*}

where *c* is the speed of light, ${T}_p$ is the radar sweep duration, *B* is the radar’s operating bandwidth, ${K}_a$ is the azimuth frequency modulation rate, *v* is the velocity of the radar platform, ${f}_m$ is the intra-pulse modulation frequency, and ${f}_a$ is the inter-pulse modulation frequency. A detailed derivation is provided in the Supplementary Information.

### Deep learning model and training

The vision-to-sequence compiler is implemented as an encoder-decoder network. The encoder contains three convolutional blocks with 32, 64, and 128 channels, respectively, each followed by ReLU activation and 2 × 2 max pooling, and converts a 501 × 401 illusion image into a 128-dimensional latent feature vector. The decoder is a two-layer LSTM with 256 hidden units per layer and generates the 141-element modulation unit sequence sequentially (Note S6). Each discrete modulation unit label is first embedded in a 64-dimensional space. The network is trained on 77 500 pairs of randomly generated sequences and simulated SAR images using the Adam optimizer (${\beta }_1$ = 0.9, ${\beta }_2$ = 0.999, $\varepsilon $ = 10⁻⁸) with a learning rate of 0.001 and a cross-entropy loss. Teacher forcing with probability 0.5 is used during training, and performance is evaluated on a hold-out test set of 2500 samples. The training process was completed in approximately 48 h on a single NVIDIA A100 GPU.

The loss function is the cross-entropy loss, defined as:


(7) \begin{eqnarray*}
\textit{Loss} = \frac{1}{{{N}_L}}\mathop \sum \limits_{t = 1}^{{N}_L} CE( {{o}_t,{y}_t} ),
\end{eqnarray*}

where ${o}_t\in {\mathbb{R}}^{\textit{batch}\ \times \ 10}$ is the predicted probability distribution for the modulation unit at the *t*th pulse, ${y}_t\in {\mathbb{R}}^{\textit{batch}}$ is the ground-truth modulation unit encoding at the *t*th pulse, and ${\mathrm{CE}}( {{o}_t,{y}_t} )$ is the cross-entropy term: ${\mathrm{CE}}( {{o}_t,{y}_t} ) = - \mathop \sum \nolimits_{i = 1}^{10} {y}_{t,i}\log ( {{o}_{t,i}} )$.

### Experimental platform

The SAR imaging system consists of a VNA (Ceyear 3674D), two dual-ridged horn antennas, and a programmable three-dimensional guide rail, which positions the antennas relative to the metasurface. The system operates in a stop-and-go mode over a total length of $L\ $= 1.4 m with a step size of Δ*l* = 0.01 m, resulting in ${N}_L$= 141 measurement positions. For each position, the VNA sweeps from 5.5 to 7.5 GHz (*B* = 2 GHz). The signal duration per sweep was ${T}_p$ = 1002 ms. The SAR image was reconstructed using the range-Doppler algorithm. Reliability verification of the system can be found in Note S7. The FPGA controller synchronizes the metasurface modulation with the pulse triggers of the VNA.

## Supplementary Material

nwag263_Supplemental_File

## Data Availability

The data that support the plots within this paper and other findings of this study are available from the corresponding author upon reasonable request.
