# Supplementary material for: Deep learning-enabled temporal sequencing of metasurface for rewritable and customizable electromagnetic illusions
Source: Natl Sci Rev. 2026 May 8;13(11):nwag263. doi: 10.1093/nsr/nwag263 (PMC13270975; doi:10.1093/nsr/nwag263)
Supplement: nwag263_Supplemental_File [file nwag263_supplemental_file.pdf]

Supplementary Information for

Deep Learning-Enabled Temporal Sequencing of Metasurface for Rewritable and Customizable Electromagnetic Illusions

Haoran Han[1,2,3], Jiwei Zhao[1,2,3*], Huan Lu[1,2,3*], Yongqiang Liu[4], Bin Zheng[1,2,3*], Rongrong Zhu[5], and Hongsheng Chen[1,2,3*]

[1] *Interdisciplinary Center for Quantum Information, State Key Laboratory of Modern Optical Instrumentation, ZJU-Hangzhou Global Scientific and Technological Innovation Center, Zhejiang University, Hangzhou 310027, China*
[2] *International Joint Innovation Center, Key Lab. of Advanced Micro/Nano Electronic Devices & Smart Systems of Zhejiang, The Electromagnetics Academy at Zhejiang University, Zhejiang University, Haining 314400, China*
[3] *Zhejiang Key Laboratory of Intelligent Electromagnetic Control and Advanced Electronic Integration, State Key Laboratory of Extreme Photonics and Instrumentation, Zhejiang University, Hangzhou 310027, China*
[4] *National Key Laboratory of Scattering and Radiation, Beijing 100854, China*
[5] *School of Information and Electrical Engineering, Hangzhou City University, Zhejiang 310015, China*

***Corresponding Author**: jackokie@zju.edu.cn, zhengbin@zju.edu.cn, hansomchen@zju.edu.cn*

The PDF file includes:

Supplementary Note S1: Feeding Structure and Measurement Method for the Amplitude-Coding Time-Modulated Metasurface 2
Supplementary Note S2: Theoretical Derivation of Spectrum Manipulation by Time-Modulated Metasurfaces 4
Supplementary Note S3: Mathematical Modeling of Illusionary Replica Generation in 1D Range Profiles Using Time-Modulated Metasurfaces 6
Supplementary Note S4: Measurement Method for the One-Dimensional Range Profile 9
Supplementary Note S5: Mathematical model for the generation of illusionary replicas in two-dimensional SAR images 10
Supplementary Note S6: Deep learning-based end-to-end model 14
Supplementary Note S7: Model Training 17
Supplementary Note S8: Post-training model performance verification 20
Supplementary Note S9: Architecture and imaging performance of the rail SAR system 28
Supplementary Note S10: Validation of similarity between simulation and experimental results 30
Supplementary Note S11: Expansion of temporal modulation waveforms based on 1 bit amplitude coding metasurfaces 32
Supplementary Note S12: Evaluation of illusion generation performance 34
Supplementary Note S13: Elucidation of the gene inspired modulation architecture and biomimetic mapping 37
Supplementary Note S14: Supplementary explanation of the Methods section 40
References 42

Supplementary Note S1: Feeding Structure and Measurement Method for the Amplitude-Coding Time-Modulated Metasurface

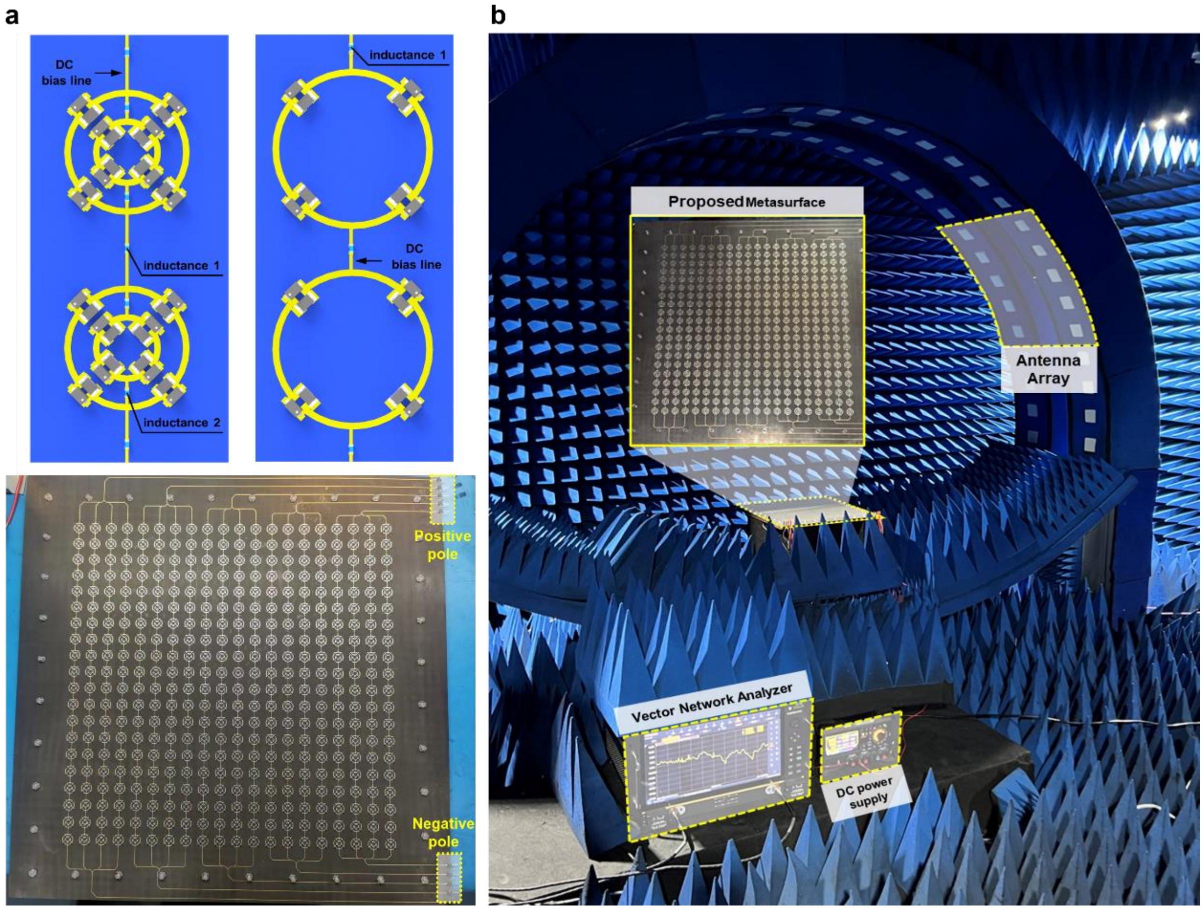

Figure S1. Structural details and measurement methodology for the amplitude-coding metasurface. **a,** Schematic of the voltage control scheme. Each column of the metasurface elements is biased in series via a surface feed line, enabling column-wise voltage control. **b,** The experimental setup for measuring the reflection coefficient amplitude (S_{11}) of the metasurface. The measurement is performed within an anechoic chamber using a vector network analyzer (VNA) and an arc-shaped gantry system, under various applied DC bias voltages.

In order to realize a metasurface with a tunable reflection coefficient amplitude, the unit cell is meticulously designed. Specifically, at each of the four gaps located on the inner and outer metallic rings of the first-layer patch, a pair of components is loaded in a parallel configuration: one PIN diode (Model: SMP1320-079LF) and one 130 Ω chip resistor. The primary function of the PIN diode is to act as a switch, whose ON and OFF conducting states are actively controlled by an external bias voltage [S1].

To facilitate this control, a direct current (DC) biasing network, implemented in the form of surface feed lines, is adopted, as schematically depicted in Figure S1a. Within this network, all the unit cells in each column of the metasurface are connected in series, allowing a common bias voltage to be applied to them simultaneously. Furthermore, to ensure the integrity of both the radio frequency (RF) performance and the DC biasing, inductors are strategically incorporated to function as RF chokes. This serves to provide effective RF-DC isolation and mitigate any potential interference between the high-frequency operational signals and the low-frequency control circuit.

Specifically, a first inductor, $L_1 = 8.2\ nH$, is inserted into the biasing line between two adjacent unit cells, and a second inductor, $L_2 = 12\ nH$, is integrated between the inner and outer metallic rings of the top-layer unit cell.

The experimental validation and performance characterization of the designed metasurface system are conducted within a microwave anechoic chamber. The measurement setup, as illustrated in Figure S1b, employed three main pieces of equipment: a vector network analyzer (VNA), a programmable DC power supply, and transmitting and receiving horn antennas.

The measurement procedure involves a coordinated operation of this equipment. The role of the programmable DC power supply is to provide and precisely set the bias voltage applied to the metasurface, thereby establishing a specific operational state. For each distinct voltage level supplied by the power source, the vector network analyzer, in conjunction with the transmitting and receiving antennas, performs a measurement to acquire the reflection coefficient amplitude curve of the metasurface. This process is systematically repeated across a range of different bias voltages to fully characterize how the reflective properties of the metasurface respond to the voltage control, thus confirming its tunable functionality.

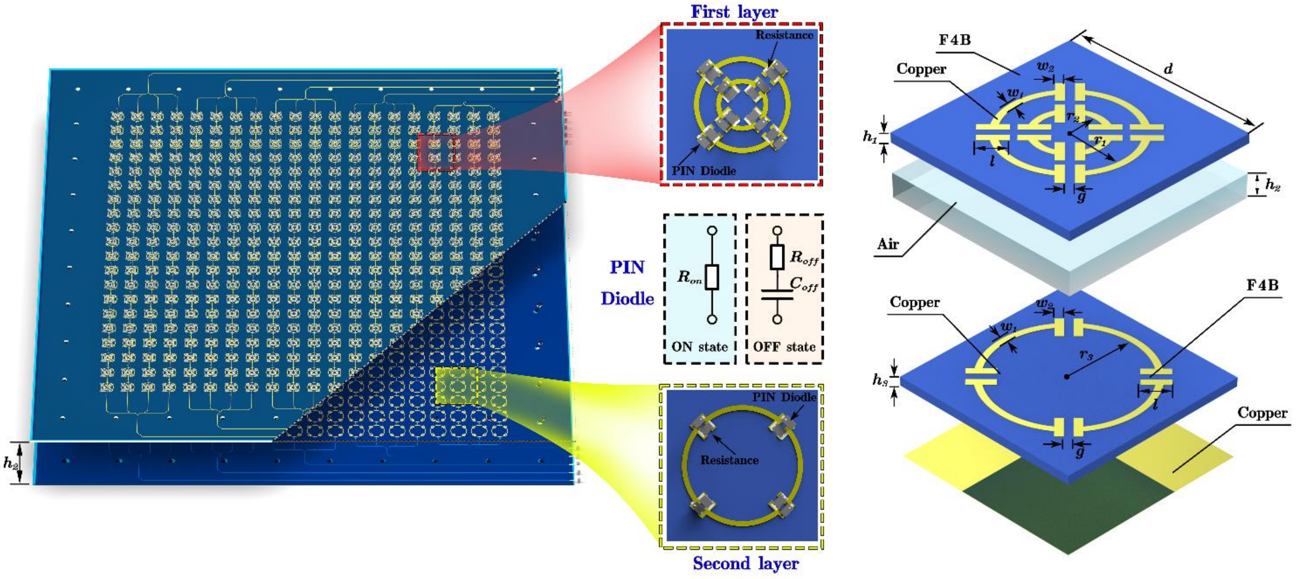

Figure S2. Schematic of the amplitude-programmable metasurface unit cell with structural parameters $d = 15\ mm$, $r_1 = 4.30mm$, $r_2 = 2.10mm$, $w_2 = 0.6\ mm$, $w_1 = 0.5\ mm$, $g = 0.6\ mm$, $l = 2\ mm$, $r_3 = 5.5\ mm$. The equivalent circuit model of the PIN diode is characterized by $R_{on} = 1\ \Omega$ in the ON state and by $R_{off} = 1000\ \Omega$ and $C_{off} = 0.23\ pF$ in the OFF state.

Supplementary Note S2: Theoretical Derivation of Spectrum Manipulation by Time-Modulated Metasurfaces

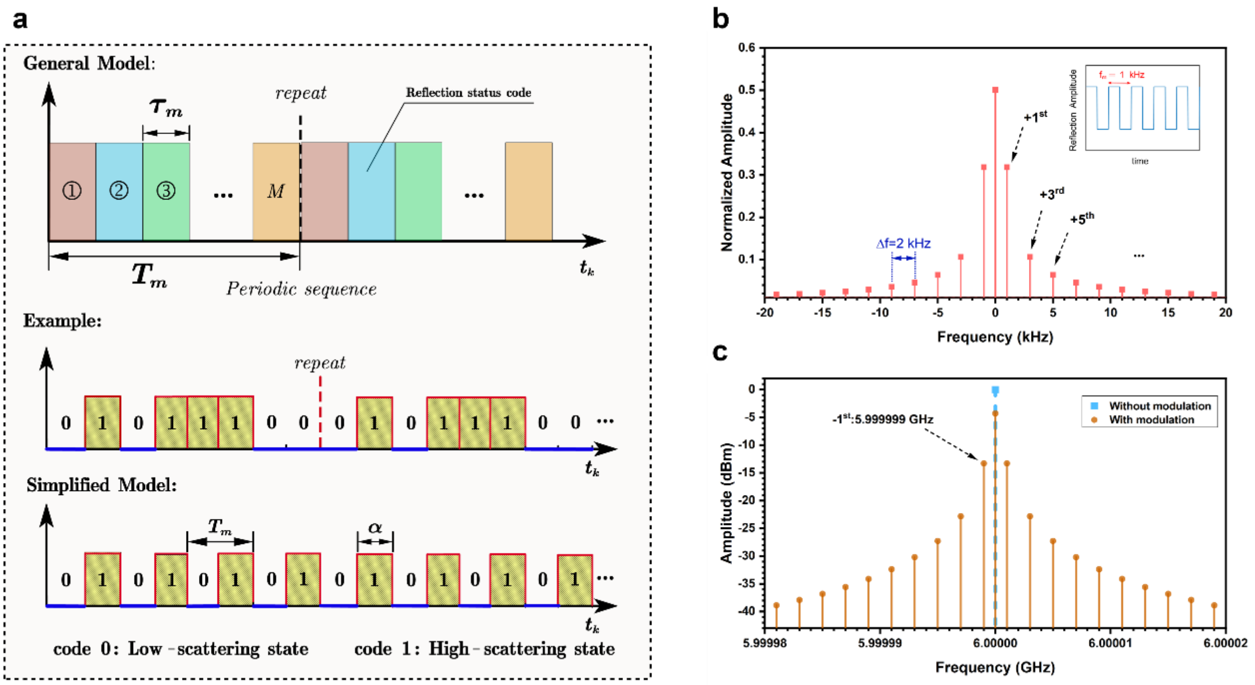

Figure S3. Mathematical modeling of time modulation. **a,** Schematic of the time-varying reflection coefficient $\Gamma(t)$ for different control waveforms (e.g., square wave and periodic sequence). **b,** Calculated spectrum of the reflection coefficient $\Gamma(f)$ under square-wave modulation. **c,** Calculated spectrum of the echo wave for a 6 GHz incident signal on the square-wave modulated metasurface.

For the reflective metasurface proposed in this paper, we begin with the assumption that all unit cells exhibit an identical reflection coefficient at any given moment [S2] . This reflection coefficient, however, varies with time and can be denoted as $\Gamma(t)$. When a plane wave $E_i(t)$, is normally incident upon the metasurface, the reflected wave $E_r(t)$ can be expressed as the product of the incident wave and the time-varying reflection coefficient:

$$E_r(t) = E_i(t)\Gamma(t) \tag{S1}$$

As illustrated in Figure S3a, and without loss of generality, we assume that the variation of the reflection coefficient's amplitude is a periodic function with a period of T_m. This period is divided into M discrete time slots of equal duration. The reflection coefficient's amplitude within the m-th time slot is denoted as Γ_m. Consequently, this time-varying function can be mathematically represented as:

$$\Gamma(t) = \sum_{m=0}^{M-1} \Gamma_m g(t - m\tau), (0 \le t < T_m) \tag{S2}$$

Here, $g(t)$ is a periodic rectangular pulse function with a pulse width of $\tau = T_m/M$. The function $g(t)$ can be expanded using its Fourier series:

$$g(t) = \sum_{k=-\infty}^{\infty} c_k \exp\left(jk\frac{2\pi}{T_m}t\right) = \sum_{k=-\infty}^{\infty} c_k \exp(jkf_m t) \tag{S3}$$

where $f_m = 1/T_m$ is defined as the modulation frequency, and c_k represents the Fourier series coefficients.

By substituting the series for $g(t)$, the Fourier series expansion of the time-varying reflection coefficient $\Gamma(t)$ is derived as:

$$\Gamma(t) = \sum_{k=-\infty}^{\infty} \left[\frac{1}{M}\operatorname{sinc}\left(\frac{k}{M}\right)\exp\left(-j\frac{k\pi}{M}\right)\left(\sum_{m=0}^{M-1}\Gamma_m \exp\left(-jk\frac{2m\pi}{M}\right)\right)\right]\exp(jk2\pi f_m t) \tag{S4}$$

Its corresponding frequency spectrum can then be expressed as:

$$\Gamma(f) = \sum_{k=-\infty}^{\infty} \left[\frac{1}{M}\operatorname{sinc}\left(\frac{k}{M}\right)\exp\left(-j\frac{k\pi}{M}\right)\left(\sum_{m=0}^{M-1}\Gamma_m \exp\left(-jk\frac{2m\pi}{M}\right)\right)\right]\delta(f - kf_m) \tag{S5}$$

According to the convolution theorem, multiplication in the time domain is equivalent to convolution in the frequency domain. Therefore, the spectrum of the reflected wave is given by the convolution of the incident spectrum and the reflection coefficient's spectrum:

$$E_r(f) = E_i(f) * \Gamma(f) \tag{S6}$$

The derivation above leads to a significant conclusion: the spectrum of the reflected wave is effectively the spectrum of the reflection coefficient shifted to the frequency of the incident wave. This directly implies that control over the reflection coefficient's spectrum is equivalent to control over the reflected wave's spectrum. Thus, it is theoretically confirmed that time-domain coding metasurfaces possess the capability to manipulate the spectrum of electromagnetic waves.

As a special case, consider the scenario depicted in Fig. 2b, where $M = 2$, $f_m = 1\ kHz$, $\Gamma_0 = 0$ and $\Gamma_1 = 1$. For this configuration, the Fourier series for the reflection coefficient simplifies to:

$$\Gamma(t) = \sum_{k=-\infty}^{\infty} \left[\frac{1}{2}\operatorname{sinc}\left(\frac{k}{2}\right) exp\left(-j\frac{3k\pi}{2}\right)\right]\exp(jk2\pi f_m t) \tag{S7}$$

The corresponding spectrum is:

$$\Gamma(f) = \sum_{k=-\infty}^{\infty} \left[\frac{1}{2}\operatorname{sinc}\left(\frac{k}{2}\right)\exp\left(-j\frac{3k\pi}{2}\right)\right]\delta(f - kf_m) \tag{S8}$$

Due to the mathematical properties of the Sinc function, the term $sinc\left(\frac{k\pi}{2}\right)$ evaluates to zero for all non-zero even integers of k. Consequently, all even-order harmonics are suppressed, and only the fundamental (carrier) and odd-order harmonics appear in the spectrum. This theoretical result is validated by the calculation results from MATLAB, as shown in Figure S3b. Furthermore, through theoretical calculation, when the incident wave's frequency is 6 GHz, its resulting spectrum is as shown in Figure S3c. The frequency spacing between the adjacent odd-order harmonics is $\Delta f = 2\ kHz$.

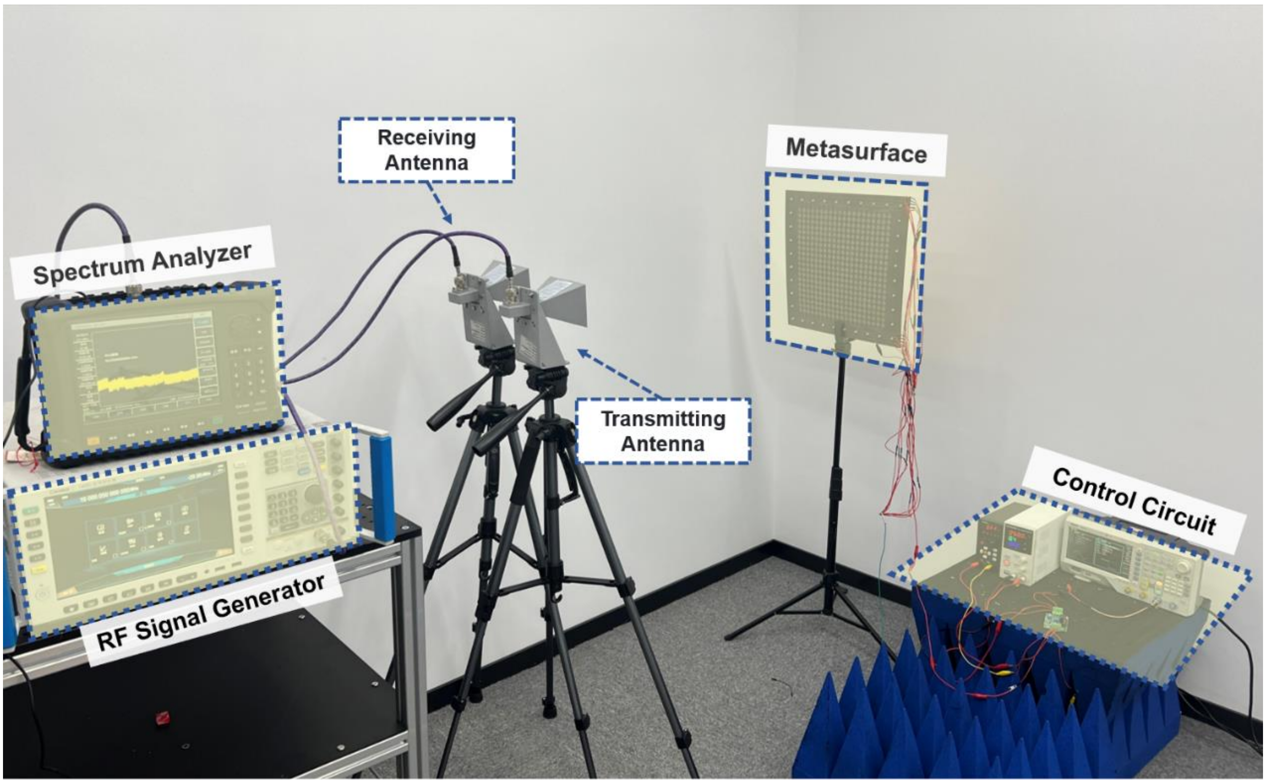

Figure S4. Experimental setup for measuring the spectrum control performance of the time-modulated metasurface.

As illustrated in Figure S4, a proof-of-concept continuous-wave (CW) radar system is constructed to experimentally verify the preceding theoretical derivations. This radar system comprises a vector signal generator (VSG), a spectrum analyzer, and a pair of transmitting and receiving antennas. During the experiment, the proposed metasurface is positioned 2 m directly in front of the horn antennas, a configuration that ensures the electromagnetic waves are normally incident upon the metasurface.

The control system for the metasurface is composed of three main parts: a Field-Programmable Gate Array (FPGA), a voltage amplification circuit, and a regulated DC power supply. The FPGA, serving as the core of this system, is responsible for generating the precise voltage control signals at specific frequencies. These signals are then amplified by the voltage amplification circuit to a level sufficient to drive the switching of the PIN diodes between their ON and OFF states, thereby dynamically modulating the reflective properties of the metasurface.

In the measurement procedure, the vector signal generator continuously transmits a 6 GHz CW signal, which illuminates the metasurface. The spectrum analyzer, connected to the receiving antenna, is then used to measure the spectrum of the echo wave reflected from the metasurface. This measurement is performed under various conditions, corresponding to the different modulation signals applied to the metasurface by its control system.

Supplementary Note S3: Mathematical Modeling of Illusionary Replica Generation in 1D Range Profiles Using Time-Modulated Metasurfaces

In the context of Synthetic Aperture Radar (SAR), after each pulse is transmitted, the echo signal received by the radar system undergoes processing steps such as mixing and matched filtering to form a one-dimensional (1D)

range profile [S6],[S7]. This range profile reflects the scattering characteristics of the target along the range direction. A critical step in this process is the application of matched filtering to the echo signal. The core objective of matched filtering is to maximize the signal energy concentration by maximizing the similarity between the received echo and the transmitted waveform, thereby achieving high-resolution imaging. Consequently, the matched filter not only determines the resolution and sidelobe characteristics of the range profile but also directly influences the accurate reconstruction of target features during the imaging process.

Therefore, an analysis of the interaction mechanism between the signal from a time-modulated metasurface and the matched filter is essential. Such an analysis can reveal the underlying physical principles causing the generation of ghost targets in the 1D range profile, and it also provides a theoretical foundation and engineering guidance for the subsequent design of controllable illusionary replicas.

The transmitted signal of a SAR system is typically a Linear Frequency Modulated (LFM) signal, which can be expressed as:

$$s(\hat{t}, t_m) = \mathrm{rect}\left(\frac{\hat{t}}{T_p}\right)\exp\left[j2\pi\left(f_c\hat{t} + \frac{1}{2}K_r\hat{t}^2\right)\right] \tag{S9}$$

Here, $\hat{t}$ represents the fast time within the radar pulse, corresponding to the SAR range direction, while t_m represents the slow time between pulses, corresponding to the SAR azimuth direction. T_p denotes the pulse width, f_c is the carrier frequency, and K_r is the chirp rate of the LFM signal. After the echo signal enters the radar receiver, it is processed through mixing and band-pass filtering to obtain the baseband signal, which can be written as:

$$r(\hat{t}, t_m) = \mathrm{rect}\left(\frac{\hat{t}}{T_p}\right)\mathrm{rect}\left(\frac{t_m}{T_L}\right)\exp\left[\frac{-4\pi j}{\lambda}R_0(t_m)\right]\exp\left\{j\pi K_r\left(\hat{t} - \frac{2R_0(t_m)}{c}\right)^2\right\} \tag{S10}$$

First, let us consider the case where the metasurface is not time-modulated. In this static scenario, its effect on the signal is a simple state of reflection or absorption, corresponding to a reflection coefficient of $\mathit{\Gamma} = 1$ or $\mathit{\Gamma} = 0$, respectively. The echo can thus be expressed as:

$$r(\hat{t}, t_m) = \mathit{\Gamma}\cdot\mathrm{rect}\left(\frac{\hat{t}}{T_p}\right)\mathrm{rect}\left(\frac{t_m}{T_L}\right)\exp\left[\frac{-4\pi j}{\lambda}R_0(t_m)\right]\exp\left\{j\pi K_r\left(\hat{t} - \frac{2R_0(t_m)}{c}\right)^2\right\} \tag{S11}$$

The time-domain expression for the matched filter in the fast-time domain is:

$$h(\hat{t}) = \mathrm{rect}\left(\frac{\hat{t}}{T_p}\right)\exp[-j\pi K_r\hat{t}^2] \tag{S12}$$

Therefore, the baseband echo signal after being processed by the matched filter can be represented by the convolution:

$$I_r(\hat{t}, t_m) = r(\hat{t}, t_m) * h(\hat{t}) \tag{S13}$$

Based on the definition of convolution, this can be further expressed as the integral:

$$I_r(\hat{t}, t_m) = \int_{-\infty}^{+\infty}\mathrm{rect}\left(\frac{u}{T_p}\right)\exp\left\{j\pi K_r\left(u - \frac{2R_0(t_m)}{c}\right)^2\right\}\mathrm{rect}\left(\frac{\hat{t}-u}{T_p}\right)\exp[-j\pi K_r(\hat{t}-u)^2] \tag{S14}$$

By solving the above expression, the final output for the 1D range profile is obtained:

$$I_r(\hat{t}, t_m) = \mathit{\Gamma}\cdot\mathrm{sinc}\left[K_rT_p\left(\hat{t} - \frac{2R_0(t_m)}{c}\right)\right]\cdot\mathrm{rect}\left(\frac{t_m}{T_L}\right)\exp\left(-j\frac{4\pi}{\lambda}R_0(t_m)\right) \tag{S15}$$

This result indicates that when $\mathit{\Gamma} = 1$, a peak corresponding to the target's 1D range profile appears at the

position defined by $R_0(t_m)$, characteristic of the Sinc function's envelope. Conversely, when $\Gamma = 0$, the target is effectively cancelled or erased from the 1D range profile.

Now, we extend the analysis to consider the mechanism and effect of a time-modulated metasurface. Consistent with the previous section, the reflection coefficient of the time-modulated metasurface can be expressed as:

$$\Gamma(t) = \sum_{m=0}^{M-1} \Gamma_m g(t - m\tau), (0 \le t < T_m) \tag{S16}$$

As derived in the preceding section, this modulation will generate a series of spectral components at specific frequency intervals. When the signal modulated by the metasurface is received by the radar, it first passes through the receiver's band-pass filter (BPF). The passband of this BPF is designed to match the spectral range of the LFM signal, which can be denoted as $\left[f_c - \frac{B}{2}, f_c + \frac{B}{2}\right]$, where B is the signal bandwidth. After modulation by the metasurface, the spectral components closest to the carrier frequency are located at $f_c \pm f_m$. Therefore, if the lower frequency limit of the modulated sideband $f_c + f_m - \frac{B}{2}$ is greater than the upper frequency limit of the BPF $f_c + \frac{B}{2}$, then all the generated sidebands will fall outside the BPF's passband. This condition $f_m > B$ means that no illusionary replicas will be observed.

Consequently, we focus on the case where $f_m < B$, the modulation frequency falls within the bandwidth of the BPF. In this scenario, the baseband signal of the radar echo after being subjected to this interference can be expressed as:

$$r_j(\hat{t}, t_m) = r(\hat{t}, t_m) \cdot \left[\sum_{n=-\infty}^{+\infty} A_n \exp(j2\pi n f_m \hat{t})\right] \tag{S17}$$

Here, $\sum_{n=-\infty}^{+\infty} A_n \exp(j2\pi n f_m \hat{t})$ is the Fourier series representation of the modulation signal, where A_n are the Fourier coefficients, *n* is the order of the harmonic, and f_m is the modulation frequency.

Next, applying the matched filter in the fast-time domain to this signal yields:

$$I_r(\hat{t}, t_m) = \sum_{n=-\infty}^{+\infty} A_n T_p \left(1 - \left|\frac{\hat{t}}{T_p}\right|\right) \exp[\varphi_r(t_m)] \mathrm{sinc}\left[K_r T_p \left(1 - \left|\frac{\hat{t}}{T_p}\right|\right)\left(\hat{t} - \frac{2R_0(t_m)}{c} + \frac{n f_m}{K_r}\right)\right] \tag{S18}$$

where $\varphi_r(t_m) = \frac{-4\pi j}{\lambda} R_0(t_m) + j2\pi n f_m \hat{t}$ is the phase of the signal after range matched filtering.

According to the properties of the Sinc function's envelope, the peak locations occur at $\hat{t} = \frac{2R_0(t_m)}{c} - \frac{n f_m}{K_r}$. Given that $R_0(t_m)$ is the slant range between the radar and the target, this result shows that the illusionary replicas will be distributed around the real target according to a specific pattern:

$$\hat{t} = \frac{2}{c}\left[R_0(t_m) - \frac{c n f_m}{2K_r}\right] \tag{S19}$$

The separation distance Δr_1 between the *n*-th order illusionary replica and the real target can be solved for as:

$$\Delta r_1 = \frac{c n f_m T_p}{2B} \tag{S20}$$

Similarly, the distance separation Δr_2 between any two illusionary replicas of arbitrary orders n_1 and n_2 is:

$$\Delta r_2 = \frac{c|n_1 - n_2| f_m T_p}{2B} \tag{S21}$$

Supplementary Note S4: Measurement Method for the One-Dimensional Range Profile

To experimentally validate the interaction mechanism between the signal modulated by the time-modulated metasurface and the matched filter, a laboratory-based Frequency-Modulated Continuous-Wave (FMCW) radar system is constructed using a vector network analyzer (VNA). The primary objective of this experiment is to verify the generation of illusionary replicas in the one-dimensional (1D) range profile when probed by this system.

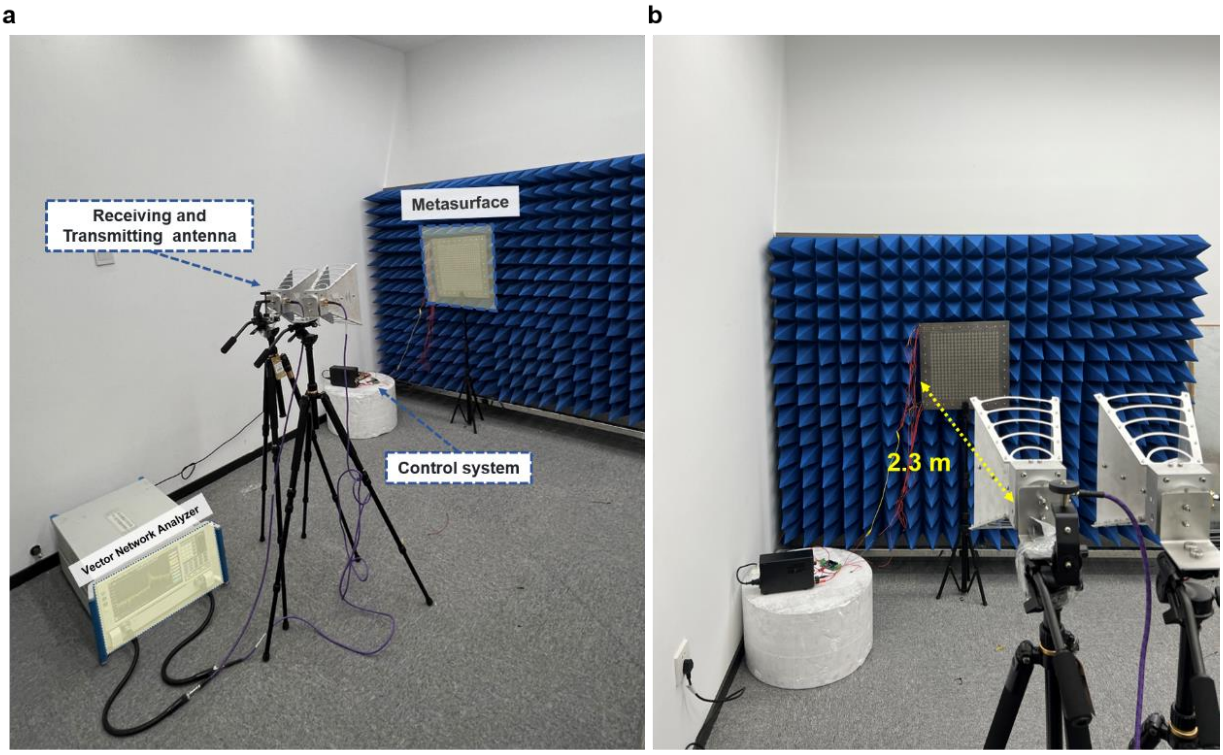

Figure S5. Experimental setup for the 1D range profile measurement. **a,** Schematic of the overall system. **b,** Front view of the setup, showing the metasurface positioned 2.3 m in front of the transmitting (Tx) and receiving (Rx) horn antennas.

As illustrated in Figure S5, the experimental system consists of a vector network analyzer, a transmitting horn antenna, a receiving horn antenna, and connecting coaxial cables. The VNA serves as the core instrument, responsible for signal transmission, reception, and processing. For this experiment, the VNA is configured with the following parameters: a frequency sweep range from 5.5 GHz to 7.5 GHz, yielding a bandwidth of $B = 2$ GHz; a total of 801 sweep points; and a sweep time of $T_p = 1002$ ms.

The experimental procedure commences with a baseline calibration measurement. First, the time-modulated metasurface is operated in a static, fully reflective state, with no modulating voltage signal applied. In this initial configuration, the actual physical distance between the metasurface and the antennas is measured. Subsequently, various distinct voltage control signals are applied to modulate the metasurface. For each corresponding modulation scheme, the resulting 1D range profile is measured by the system and stored for subsequent analysis.

Supplementary Note S5: Mathematical model for the generation of illusionary replicas in two-dimensional SAR images

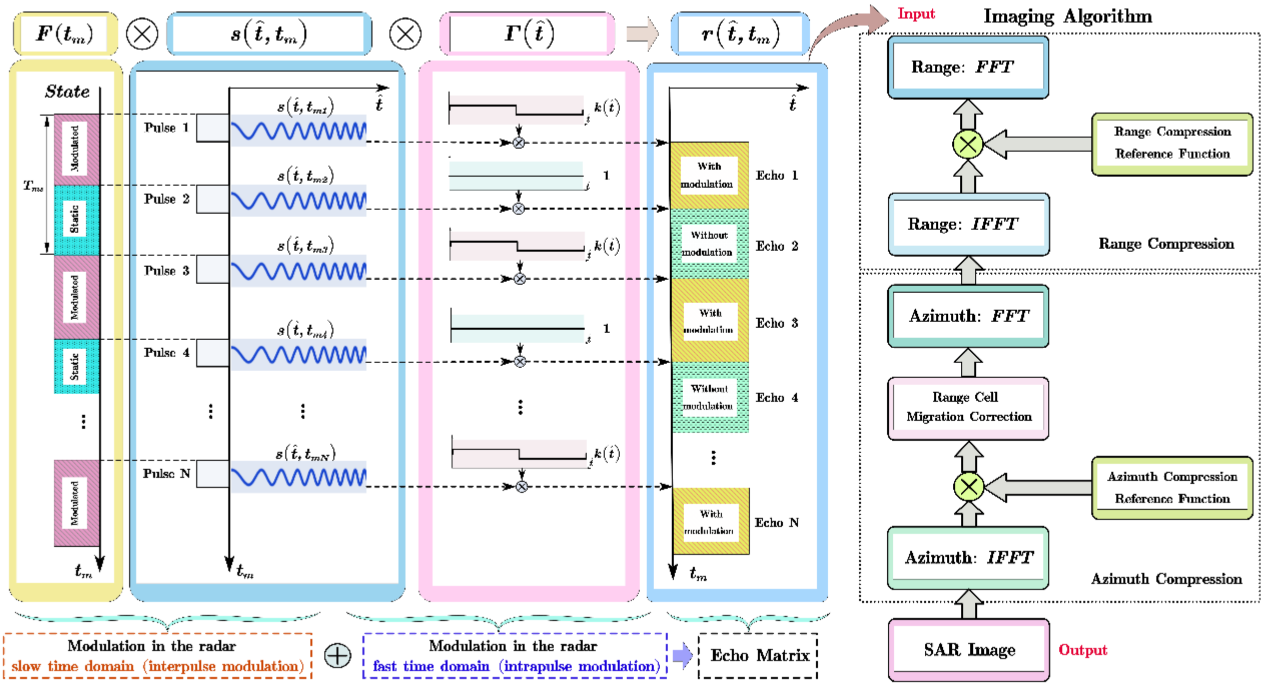

Figure S6. Flowchart of the algorithm for joint 2D illusionary replica generation and radar imaging using a genetically-inspired modulation method for the time-modulated metasurface.

Taking Synthetic Aperture Radar (SAR) as an example, to achieve high-resolution imaging, the radar system scans a target area through the platform's motion. This process involves progressively acquiring echo data covering different spatial positions to complete the two-dimensional (2D) imaging. Within the SAR system, the direction of the radar's motion is defined as the azimuth direction, or the slow-time domain. It reflects the observation process of the target from different positions of the radar platform in space. Conversely, the direction along which the radar detects ground targets via the propagation of its transmitted pulses is defined as the range direction, or the fast-time domain [S8],[S9]. This corresponds to the distance information reflected by the propagation delay between the radar and the ground target. Consequently, SAR echo data possesses a characteristic 2D structure, corresponding to the signal variations in both the azimuth and range dimensions.

This paper employs a method analogous to a genetic combination, which achieves diversified control over the echo signals by applying different types of metasurface modulations within different radar pulses. This method not only introduces modulation in the range direction (intra-pulse) but also creates an effective inter-pulse modulation in the azimuth direction through variations in the control signal between pulses. This dual modulation thereby enables the controllable generation of multi-dimensional illusionary replicas in the final SAR image. In the following, the mathematical modeling and the underlying mechanism corresponding to this method are discussed in detail.

As illustrated in the Figure S6, the core principle of SAR processing is to apply matched filtering to the SAR echo data in both the range and azimuth directions. The transmitted SAR signal is typically a Linear Frequency Modulated (LFM) waveform, which, consistent with the previous definition (Eq. (S9)), is expressed as:

$$s(\hat{t}, t_m) = \mathrm{rect}\left(\frac{\hat{t}}{T_p}\right)\exp\left[j2\pi\left(f_c\hat{t} + \frac{1}{2}K_r\hat{t}^2\right)\right] \tag{S22}$$

After being scattered by the target, the echo signal arrives at the radar receiver. Here, the echo is first mixed with a reference radio frequency (RF) signal to produce an intermediate frequency (IF) signal. This IF signal then undergoes baseband filtering to form the baseband signal. Consequently, the entire subsequent imaging process is generally performed on this baseband data. The input signal to the imaging processor at this stage can be represented as:

$$r(\hat{t}, t_m) = \mathrm{rect}\left(\frac{\hat{t}}{T_p}\right)\mathrm{rect}\left(\frac{t_m}{T_L}\right)\exp\left[\frac{-4\pi j}{\lambda}R_0(t_m)\right]\exp\left\{j\pi K_r\left(\hat{t} - \frac{2R_0(t_m)}{c}\right)^2\right\} \tag{S23}$$

For now, we disregard the time-modulation characteristics of the metasurface and assume it is in a static, highly reflective state. We establish the signal processing model using the Range-Doppler Algorithm (RDA).

According to the RDA, the first step is to perform matched filtering on the signal in the range direction, a process known as range compression. The mathematical expression for the range matched filter is:

$$h_r(\hat{t}) = \mathrm{rect}\left(\frac{\hat{t}}{T_p}\right)\exp(-j\pi K_r\hat{t}^2) \tag{S24}$$

Thus, the baseband echo signal after range matched filtering can be expressed as a convolution:

$$I_r(\hat{t}, t_m) = r(\hat{t}, t_m) * h(\hat{t}) \tag{S25}$$

Based on the definition of convolution, the result of range compression is:

$$I_r(\hat{t}, t_m) = \Gamma \cdot \mathrm{sinc}\left[K_r T_p\left(\hat{t} - \frac{2R_0(t_m)}{c}\right)\right] \cdot \mathrm{rect}\left(\frac{t_m}{T_L}\right)\exp\left(-j\frac{4\pi}{\lambda}R_0(t_m)\right) \tag{S26}$$

The second step is to apply an azimuth Fourier Transform to the range-compressed signal. Under the assumption of a low squint angle, where the radar beam points towards the zero-Doppler direction, $R_0(t_m)$ can be approximated as:

$$R_0(t_m) = \sqrt{{R_B}^2 + (vt_m)^2} \approx R_B + \frac{v^2{t_m}^2}{2R_B} \tag{S27}$$

Using the Principle of Stationary Phase (POSP), a time-frequency relationship exists in the azimuth direction, given by $f_{tm} = -K_a t_m$, with the azimuth chirp rate $K_a = \frac{2v^2}{\lambda R_B}$. Therefore, the signal after the azimuth Fourier Transform is:

$$I_2(\hat{t}, f_{tm}) = A_\sigma \mathrm{rect}\left(\frac{\hat{t}}{2T_p}\right)\mathrm{W_{am}}(f_{tm} - f_{tmc})\exp\left[\frac{-j4\pi f_c R_B}{c}\right]\mathrm{sinc}\left[K_r T_p\left(\hat{t} - \frac{2R_0(f_{tm})}{c}\right)\right] \tag{S28}$$

Here, the term $R_0(f_{tm}) \approx R_B + \frac{v^2}{2R_B}\left(\frac{f_{tm}}{K_a}\right)^2 = R_B + \frac{\lambda^2 R_B {f_{tm}}^2}{8v^2}$. The second term $\frac{\lambda^2 R_B {f_{tm}}^2}{8v^2}$ represents the Range Cell Migration (RCM) component in the Range-Doppler domain, which can be corrected using interpolation techniques.

Finally, the third step is to perform azimuth matched filtering (azimuth compression). The azimuth matched filter can be expressed as:

$$h_m(t_m) = \mathrm{rect}\left(\frac{t_m}{T_L}\right)\exp(j\pi K_a {t_m}^2) \tag{S29}$$

Consequently, the final focused image or the result of azimuth compression is:

$$I_3(\hat{t}, t_m) = A_\sigma \text{sinc}\left[K_r T_p \left(\hat{t} - \frac{2R_B}{c}\right)\right] \text{sinc}(K_a T_L t_m) \exp\left[\frac{-j4\pi f_c R_B}{c}\right] \tag{S30}$$

where T_L is the total synthetic aperture time for forming one image, and R_B is the slant range between the radar and the target at the center of the aperture.

Now, we consider the effect of the time-modulated metasurface. We constrain the intra-pulse modulation frequency to $f_m < B$. Without loss of generality, we assume the inter-pulse modulation signal follows a periodic pattern with a period of T_a. This period is divided into *N* time slots, each with a width of τ_a, where each slot contains one radar pulse repetition interval. The modulation signal within each slot is denoted as $\Gamma_n(\hat{t})$. This results in a 2D time-varying function that can be expressed as:

$$\Gamma_{2D}(\hat{t}, t_m) = \sum_{n=0}^{N-1} \Gamma_n(\hat{t}) g(t_m - n\tau_a), (0 \le t_m < T_a) \tag{S31}$$

where $\Gamma_n(\hat{t})$ represents the metasurface modulation signal corresponding to each "gene" in the sequence. Since its expression is consistent with the intra-pulse modulation model, this "genetic sequence" of modulations can be written as:

$$\Gamma_{2D}(\hat{t}, t_m) = \sum_{n=0}^{N-1} \left[\sum_{m=0}^{M-1} \Gamma_m g(t - m\tau)\right] g(t_m - n\tau_a), (0 \le t_m < T_a) \tag{S32}$$

To facilitate the discussion of how different inter-pulse modulation waveforms affect ghost target generation, we denote the inter-pulse modulation signal as $F(t_m)$ and expand it using its Fourier series:

$$F(t_m) = \sum_{q=-\infty}^{+\infty} S_q \exp(j2\pi q f_a t_m) \tag{S33}$$

where q is the order of the Fourier series harmonic, S_q is the coefficient of the q-th order harmonic, and f_a is the frequency of the inter-pulse modulation signal. Therefore, considering the combined intra-pulse and inter-pulse interference, the modified baseband radar echo is:

$$r_b(\hat{t}, t_m) = r(\hat{t}, t_m) \cdot \Gamma(t) \cdot F(t_m) \tag{S34}$$

By substituting this signal into the aforementioned imaging process, the final result is obtained:

$$I_a = \sum_{q=-\infty}^{+\infty} \sum_{k=-\infty}^{+\infty} D_{qk} \text{sinc}\left[K_r T_p \left(\hat{t} - \frac{2R_0(t_m)}{c} + \frac{kf_m}{K_r}\right)\right] \text{sinc}\left[K_a T_L \left(1 - \left|\frac{t_m}{T_L}\right|\right)\left(t_m - \frac{qf_a}{K_a}\right)\right] \exp[\varphi_a(\hat{t}, t_m)] \tag{S35}$$

where $\varphi_a(\hat{t}, t_m) = exp\left(j\pi q f_a t_m + j\pi k f_m \hat{t} - j\frac{4\pi}{\lambda} R_B\right)$, $K_a = \frac{-2v^2}{\lambda R_0}$, v is the velocity of the radar platform, and R_B is the slant range to the target from the center.

Based on the properties of the Sinc function, the peaks in the slow-time domain appear at $t_m = \frac{qf_a}{K_a}$, and the peaks in the fast-time domain appear at $\hat{t} = \frac{2R_0(t_m)}{c} - \frac{kf_m}{K_r}$. In the azimuth direction, the separation distance Δa between ghost targets of any arbitrary orders q_1 and q_2 is:

$$\Delta a = \frac{|q_1 - q_2| f_a}{K_a} \cdot v \tag{S36}$$

Through modulation in both the intra-pulse (range) and inter-pulse (azimuth) dimensions, ghost target peaks are generated at specific intervals in both range and azimuth. The precise locations of these illusionary replicas can be

calculated based on the derived equations (Eq.(S21) and Eq.(S36)).

It is imperative to explicitly clarify that the theoretical derivations and proofs detailed above are fundamentally established upon idealized mathematical derivations, rather than comprehensive full wave synthetic aperture radar electromagnetic simulations. Consequently, this simplified mathematical framework inherently possesses certain physical limitations or idealizations when compared to authentic practical scenarios. Specifically, to accelerate the data generation process and focus on the core temporal modulation mechanisms, the following three major physical effects were simplified. First, spatial propagation loss was entirely neglected, whereas in real physical systems, the radar echo intensity inherently attenuates with increasing propagation distance. Second, the metasurface was mathematically modeled as a combination of discrete point targets, with its total scattering equivalently calculated as the direct linear superposition of these multiple points, thereby ignoring complex electromagnetic coupling effects. Third, the practical antenna radiation pattern, which inevitably causes echo intensity variations at different observation angles in real world scenarios, was completely omitted. In summary, the forward echo derivation utilized in our work constitutes an idealized and simplified mathematical model, intentionally abstracted from the full physical complexities of real synthetic aperture radar imaging systems.

Supplementary Note S6: Deep learning-based end-to-end model

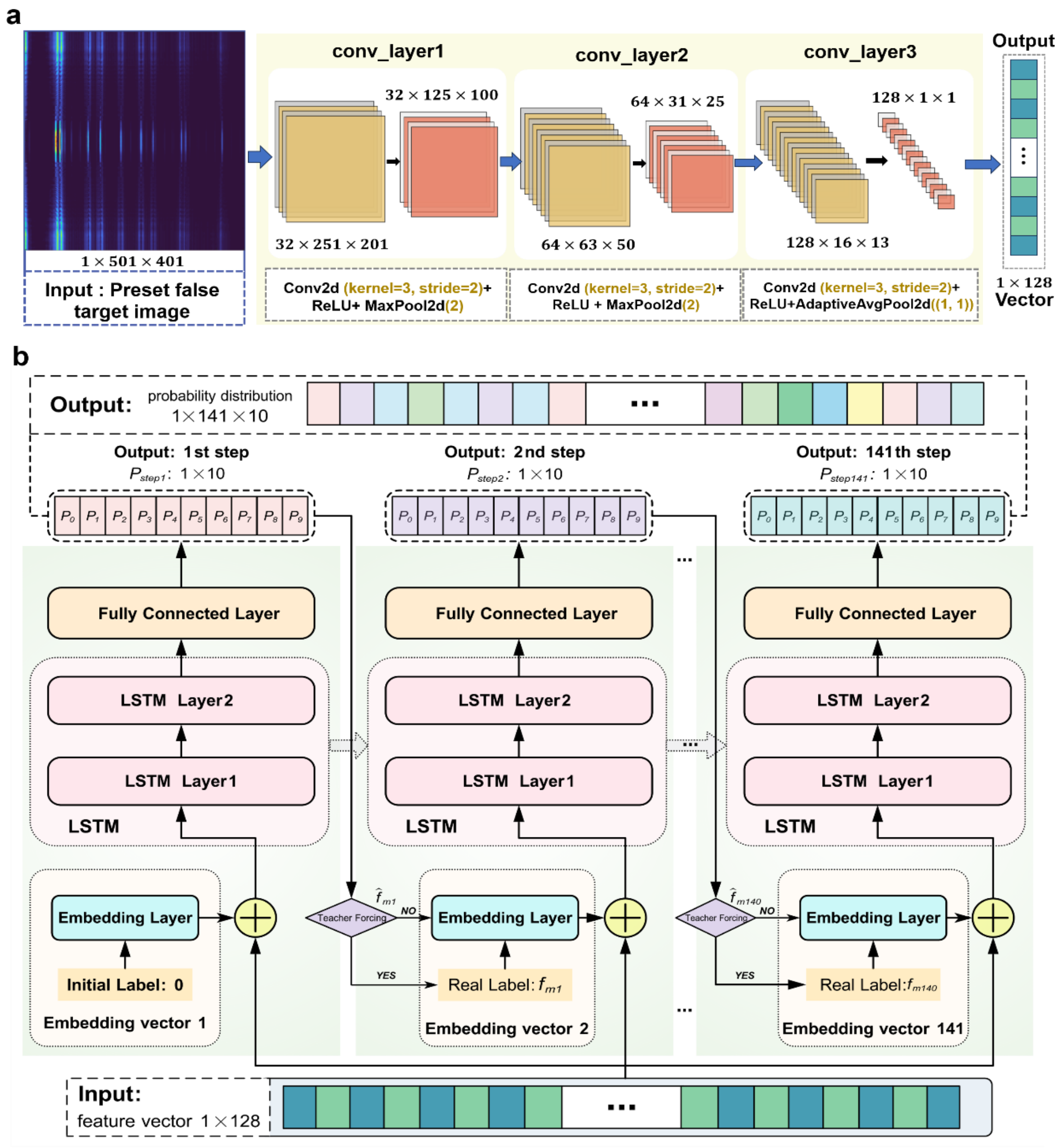

Figure S7. Architecture of the deep learning-based " illusionary replica distribution-to-modulation gene sequence" end-to-end model. **a,** Encoder architecture: A Convolutional Neural Network (CNN) that performs multi-scale feature extraction from the input illusionary replica image. **b,** Decoder architecture: A Long Short-Term Memory (LSTM) network that generates the physically realizable modulation gene sequence.

As detailed in Supplementary Note 2, applying distinct metasurface temporal modulation waveforms namely genes across different pulses generates diverse electromagnetic illusions in the synthetic aperture radar image. We formulate this forward physical model as:

$$I = F(G) \tag{S37}$$

where $G = [g_1, g_2, \dots, g_{N_L}]$ denotes the time modulation coding sequence in the slow time dimension, and I

represents the generated two dimensional image. Consistent with the experimental scenario, the pulse quantity is strictly set to $N_L = 141$. Each element g_n represents the temporal modulation waveform $\Gamma_n(\hat{t})$ of the corresponding pulse, selected from a predefined gene library containing M_{gen} waveforms. To appropriately simplify the problem space, we fix the temporal modulation waveform as a square wave with a duty cycle of 0.5, leaving the modulation frequency f_m as the sole independent variable.

The range resolution of the adopted radar is $c/(2B) = 0.075$ m, and the pulse width T_p is 1000 ms. The spatial range shift is determined by the theoretical formula:

$$\Delta R = \frac{c|k_1 - k_2|f_m T_p}{2B} \tag{S38}$$

To effectively generate a false illusion, the spatial shift must physically exceed one resolution cell, meaning $\Delta R > 0.075$ m. This specific condition dictates that the minimum modulation frequency must be greater than 2 Hz. Concurrently, the illusion generation distance cannot exceed the maximum radar detection range $R_{smax} = \pm 30$ m, which fundamentally restricts the maximum frequency limit to 400 Hz. To mitigate the influence of measurement errors during testing, we discretize the selectable frequency set as $f_m \in \{0,10,20,\ldots,90\}$ Hz. This specific configuration provides a total of 10 modulated square waves with distinct frequencies, establishing a well defined and practical task space.

Because the forward mapping is highly nonlinear and multiple modulation sequences can produce highly similar two-dimensional topological structures, a unique analytical inverse mapping strictly does not exist. Therefore, we construct an end-to-end deep learning model $M_\theta(\cdot)$ to solve this mapping relationship:

$$G_{pred} = M_\theta(I_{target}) \tag{S39}$$

where θ represents the trainable network parameters, and G_{pred} denotes the predicted metasurface gene sequence to realize the input target illusion I_{target}. The fundamental objective of this deep learning model is not to compute an exact analytical inverse, but to autonomously generate feasible modulation strategies that satisfy physical constraints.

The deep learning-based framework proposed in this paper establishes an end-to-end mapping from a "illusion image" to a "modulation gene sequence." It primarily adopts an encoder-decoder architecture. The encoder, based on a Convolutional Neural Network (CNN), performs multi-scale feature extraction from the input illusionary replica image. The decoder, utilizing a Long Short-Term Memory (LSTM) network, establishes the non-linear mapping relationship between temporal dynamic features and the modulation parameters. This process ultimately generates a physically realizable 1×141-dimensional modulation gene sequence.

Specifically, as illustrated in Figure S7a, the encoder section employs a hierarchical CNN architecture to achieve feature abstraction and compression from the illusionary replica distribution image through multi-level convolution and pooling operations [S12][S13]. This section is composed of three cascaded convolutional units, with each unit comprising a convolutional layer, an activation function, and a pooling layer.

Initial Feature Extraction Layer: This layer uses a combination of a 32-channel, 3×3 convolution and a 2×2 max-pooling operation. It compresses the input image dimensions to 125×100, achieving preliminary feature dimensionality reduction and optimizing computational efficiency through low-dimensional space mapping.

Intermediate Feature Abstraction Layer: This stage is based on a cascaded structure of 64-channel, 3×3

convolutions, which further reduces the output feature map dimensions to 63×50.

High-Level Feature Compression Layer: This final layer integrates a 128-channel, 3×3 convolution with an adaptive global average pooling layer. It ultimately outputs a 128-dimensional global vector, providing a high-information-density spatial context as input for the decoder module.

This hierarchical design effectively compresses the image information while preserving the key features necessary for the subsequent decoder to utilize.

As shown in Figure S7b, the decoder section utilizes an LSTM-based module for generating the gene sequence. In its specific implementation, the gene class label—either from the ground truth or the network's prediction at the previous step—is first passed through an embedding layer. This layer maps the input into a 64-dimensional vector space, a technique that effectively avoids the curse of dimensionality associated with one-hot encoding. Subsequently, this embedding vector is concatenated with the 128-dimensional feature vector output by the encoder, forming the complete input for the LSTM at the current time step. This concatenation method ensures that the global image features extracted by the encoder continuously participate in the prediction at every time step, thereby enhancing the overall consistency and accuracy of the generated sequence.

The core of the decoder is a two-layer stacked LSTM network, with the number of hidden units set to 256. The network operates in a batch-first mode. During the training process, a Teacher Forcing strategy is introduced, which uses the ground-truth label as the input for the next time step with a certain probability. This approach accelerates model convergence and mitigates the problem of error accumulation. In this work, the forcing probability is set to $P_{force} = 0.5$. With probability P_{force}, the true modulation frequency gene is injected to enhance sequential continuity learning; with probability $1 - P_{force}$, the self-generated frequency gene is used to improve its generalization capability.

At each time step $t \in \{1,2,\dots,141\}$, the top-layer LSTM outputs a 256-dimensional hidden state. This state is passed through a fully connected layer, which maps it to a 10-dimensional probability distribution. This distribution corresponds to the probabilities of the 10 possible frequency modulation genes, denoted as $\mathrm{O} = [\mathrm{o}_1, \mathrm{o}_2, \dots, \mathrm{o}_{141}] \in \mathbb{R}^{batch\times 141\times 10}$. An argmax operation is then applied to forcibly output an integer index value from 0 to 9, representing the encoding for one of the 10 modulation genes. This sequence is denoted as $G = [g_1, g_2, \dots, g_{141}] \in \mathbb{Z}^{batch\times 141}$. After a 10-fold frequency conversion, the frequency of the metasurface's square-wave control signal within each pulse is obtained. This yields the required 1×141-dimensional modulation gene sequence: $f_{m,t} = 10 \cdot g_t \in \{0,10,20,\dots,90\}$ Hz.

Table S1 Core model parameters

Parameter category	Parameter name	Value / Setting
**Encoder**	Output feature vector dimension	128
	Kernel size	3×3
	Conv layer 1 output channels	32
	Conv layer 2 output channels	64
	Conv layer 3 output channels	128
	Activation function	ReLU
**Decoder**	Embedding size	64
	LSTM hidden size	256
	LSTM layers	2
	Target sequence length	141
	Number of classes	10

Supplementary Note S7: Model Training

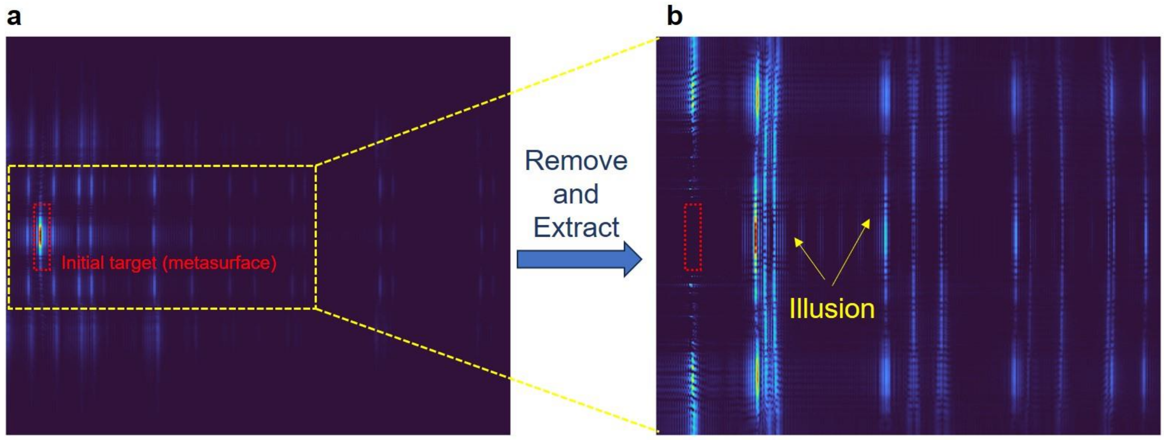

Figure S8 SAR image generation and illusion extraction. **a,** The complete SAR imaging result generated via numerical simulation in MATLAB, showing both the metasurface target at its original position and the resulting illusion. **b,** The isolated illusion image after post-processing the image in (a) to remove the original target signature.

Leveraging the theoretical computation model described previously, we can numerically simulate the Synthetic Aperture Radar (SAR) imaging process using MATLAB. This model was implemented in MATLAB based on pre-defined radar operating frequencies and scanning parameters (the detailed computational methodology is available in Supplementary Note 5). This computational framework enables the large-scale generation of diverse modulation frequency combinations, which we analogously refer to as "genotypes." Subsequently, for each "genotype" sequence, the theoretical model is used to compute the resulting illusion artifacts generated in the SAR image. This automated

approach allows for the rapid creation of a comprehensive dataset consisting of numerous one-to-one corresponding genotype-phenotype pairs.

A critical post-processing step is then applied to refine the generated images for model training. The theoretical calculations and spectrum modulation results reveal that due to an inherent fundamental wave component in the amplitude-time modulation, a residual signature of the original target persists at its initial coordinates in the SAR image. Consequently, it is necessary to remove this residual target signature to isolate the illusion data. Furthermore, to reduce data dimensionality and focus on relevant features, we crop the image to exclude extraneous regions where no illusion artifacts appear. This procedure ultimately yields a standardized image dataset with dimensions of 501×401. The entire data generation and processing workflow is illustrated in Figure S8.

The specific processing pipeline for the synthetic aperture radar data is detailed as follows. First, the raw imaging result is generated as a double precision matrix with spatial dimensions of 2803×800. To isolate the specific illusion components generated exclusively by the metasurface temporal modulation, we perform a precise background subtraction operation:

$$I_{illusion} = I_{modulated} - I_{origin} \tag{S40}$$

where $I_{modulated}$ represents the synthetic aperture radar imaging result with metasurface modulation, and I_{origin} is the baseline image without any modulation. This subtraction operation effectively extracts the pure electromagnetic contribution of the temporal modulation sequence. Subsequently, a specific sub region containing both the primary target and the generated illusion areas is spatially cropped, which further reduces the data matrix dimensions to 1000×401.

After obtaining this isolated illusion image, a linear amplitude normalization is applied. We first calculate the absolute maximum amplitude value of the image matrix:

$$I_{max} = \max(I_{illusion}) \tag{S41}$$

and subsequently normalize the entire data matrix:

$$I_{norm} = \frac{I_{illusion}}{I_{max}} \tag{S42}$$

Following this normalization step, a second spatial cropping is performed to further scale the matrix dimensions down to 500×401, strictly focusing on the localized region where the target illusion structure is situated. Finally, the float data type is mathematically converted into an 8-bit grayscale format, namely uint8, to serve as the standardized input for the subsequent deep learning neural network.

To train this model, we generate a dataset of 77,500 frequency gene sequences, each of size 1×141, using a randomized approach. For each sequence, the corresponding SAR image result is calculated via the aforementioned theoretical forward model. The illusionary replicas are then extracted from these images to form image data of size 501×401. Therefore, the dataset consists of pairs of processed SAR echo image matrices and their corresponding frequency gene encoding vectors. The SAR images have a depth of 8 bits, and each element in the frequency gene vector has a value in the range $\{0,10,20,\ldots,90\}$, which is discretized and mapped to integer gene classes from 0 to 9 for training.

Building upon the aforementioned standard processing pipeline, we deliberately introduce random perturbation mechanisms during the data generation phase to comprehensively enhance the generalization capabilities of the

model. Specifically, small amplitude Gaussian noise is artificially injected into the normalized images:

$$I' = I + \mathcal{N}(0, \sigma^2) \tag{S43}$$

where the standard deviation σ is selected within a predefined physical range. In this specific work, we strictly utilize $\sigma = 0.01$. Furthermore, we apply a random amplitude scaling operation to the image data:

$$I' = \alpha I \tag{S44}$$

where the scaling coefficient α strictly follows a uniform continuous distribution mathematically denoted as $\mathcal{U}(0.8, 1.2)$. Additionally, to simulate non ideal practical conditions, we artificially introduce weak energy distributions simulating defocusing effects around the main illusion peaks. We also incorporate minor energy smearing artifacts that are ubiquitous in practical synthetic aperture radar imaging systems, thereby substantially bolstering the robustness of the trained network.

The training process employs the Adam optimizer with a learning rate of 0.001. The loss function is the cross-entropy loss, defined as:

$$Loss = \frac{1}{N_L} \sum_{t=1}^{N_L} \mathrm{CE}(o_t, y_t) \tag{S45}$$

where $o_t \in \mathbb{R}^{batch \times 10}$ is the predicted probability distribution for the modulation gene at the *t*-th pulse, $y_t \in \mathbb{R}^{batch}$ is the ground-truth modulation gene encoding at the *t*-th pulse, and $\mathrm{CE}(o_t, y_t)$ is the cross-entropy calculation function: $\mathrm{CE}(o_t, y_t) = -\sum_{i=1}^{10} y_{t,i} \log(o_{t,i})$.

For further research, to enhance the model's generalization and stability, the volume of training data can be significantly increased. Additionally, an Attention mechanism can be introduced to improve the extraction of key features, and a Transformer architecture can be explored to further optimize long-sequence prediction. Furthermore, integrating physics-based knowledge can serve to guide the model and improve its interpretability.

Table S2 Model Training Parameters

Category	Parameter	Value / Setting
**Dataset & Dataloader**	Training samples	77,500
	Batch size	64
	Shuffle	True
**Training process**	Epochs	150
	Optimizer	Adam
	Learning rate	0.001
	Loss function	Cross-Entropy Loss (nn.CrossEntropyLoss)
**Key mechanisms**	Teacher forcing ratio	1-0
	Model saving criterion	Saves model with the highest training accuracy (best_acc)
	Checkpointing	A checkpoint is saved at the end of every epoch.

	Hardware	Utilizes CUDA-enabled GPU if available, otherwise CPU.

Supplementary Note S8: Post-training model performance verification

A. Analysis of the loss curve

In the training of our model, we selected the Cross-Entropy Loss function as the primary metric to quantify the discrepancy between the model's predictions and the ground truth labels. This function is a standard and highly effective choice for multi-class classification problems. In the context of this work, the model is required to predict a class from 10 possible categories at each position of a 141-element sequence. This task is fundamentally a series of concatenated multi-class classification challenges, making the Cross-Entropy Loss function exceptionally well-suited.

A key aspect of our methodology is the calculation of the loss value for the sequence output. Instead of a single evaluation for the entire sequence, the loss is computed individually for each time step and then averaged across the sequence length. This approach ensures that the model learns to devote equal importance to the predictive accuracy at every position, promoting a holistic understanding of the sequence.

The detailed loss calculation process per batch is as follows:

1. Element-wise Computation: For a given batch of data, the model outputs a prediction tensor with the shape [batch_size, sequence_length, num_classes], specifically [64, 141, 10]. We iterate through each time step t of the sequence (from t = 1 to t = 141).
2. Cross-Entropy Application: At each time step t, we extract the model's predictions for all samples in the batch (shape: [64, 10]) and their corresponding ground truth labels (shape: [64]). The nn.CrossEntropyLoss function is then applied to compute the loss for this specific time step.
3. Aggregation and Averaging: The loss values calculated for all 141-time steps are summed up and subsequently divided by the total sequence length (141) to obtain a final, normalized loss value for each sequence.

This element-wise averaging approach offers several distinct advantages that substantiate the effectiveness of our training regimen:

1. Ensures Sequence Integrity: By assigning equal weight to the loss at each position, the model is compelled to accurately predict the entire sequence, preventing it from prioritizing certain parts at the expense of others. This is crucial for generating functionally complete and correct sequences.
2. Provides Stable Gradients: Compared to calculating the loss only at the end of a sequence, computing it at every time step provides a richer and more stable gradient signal for optimization. This helps mitigate the vanishing gradient problem and accelerates model convergence.
3. Maintains Metric Consistency: Normalizing the total loss by the sequence length makes the metric's magnitude independent of the sequence's length. Although the length is fixed in this study, this practice ensures that performance is comparable across different models or training phases.

In summary, the adopted time-step-averaged Cross-Entropy Loss method is not only aligned with the sequential nature of our task but also mechanistically ensures the stability and comprehensiveness of the training process. This

serves as a robust foundation for driving the model toward high-precision sequence generation.

The training results affirm this approach. As depicted in the loss curves, both the training and validation sets show a clear and consistent downward trend as the number of epochs increases. The two curves converge smoothly and maintain a minimal gap, particularly in the later stages of training, which is indicative of the model's strong generalization capabilities. The best-performing model, corresponding to the lowest achieved validation loss, was saved for subsequent use. The detailed loss curves and their corresponding numerical values are provided with this paper.

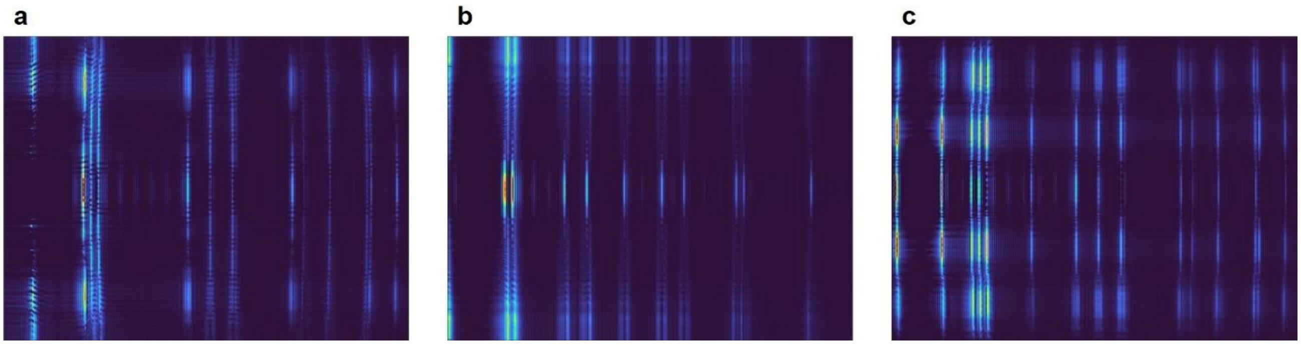

Figure S9 Preset illusion images used as input for the neural network. Panels a, b, and c correspond to three distinct test cases.

B. Validation of frequency gene sequence generation accuracy

Upon completion of training, the optimal model was selected for performance validation. To assess its generative capabilities, we created three distinct illusion patterns to serve as input images for the network. The model processed these images to yield their corresponding modulation frequency sequences, hereafter referred to as the "predicted genotypes."

It is important to note that these input illusion images were themselves originally generated by our theoretical model from pre-defined frequency sequences. These pre-defined sequences, therefore, represent the "ground truth genotypes." The validation process consists of performing an element-wise comparison between the model-predicted genotype and the ground truth genotype for each test case. The three input illusion patterns used for this validation are depicted in Figure S9a-c.

For instance, consider the case illustrated in Figure S9a. When this pre-set illusion image is fed into the trained network, it outputs a predicted control frequency sequence (the "predicted genotype"), which is presented in **Table S3** The "gene" sequence generated from the illusion in Figure S9a (read from left to right, top to bottom; size: 1×141; unit: Hz).. The original ground truth frequency sequence used to theoretically generate this illusion is shown for comparison in **Table S4** The theoretical "gene" sequence required to generate the illusion in Figure S9a (read from left to right, top to bottom; size: 1×141; unit: Hz).. An element-wise comparison between these two sequences reveals that 138 out of the 141 elements are identical. In the provided tables, mismatched elements are highlighted with blue shading for clarity. The prediction accuracy r_f for this case is calculated as $r_f = \frac{138}{141} = 97.87\%$.

Following the same procedure, the generation accuracies for the inputs shown in Figure S9b and Figure S9c were determined to be 100% and 98.58%, respectively. This yields a high average generation accuracy of 98.82%

across the three test cases, demonstrating the model's excellent fidelity and its robust ability to deduce the underlying frequency modulation from a given illusion pattern.

Table S3 The "gene" sequence generated from the illusion in Figure S9a (read from left to right, top to bottom; size: 1×141; unit: Hz).

0	70	0	70	70	90	70	80	0	70
0	70	70	90	70	80	0	70	0	70
70	90	70	80	0	70	0	70	70	90
70	80	0	70	0	70	70	90	70	80
0	70	0	70	70	80	70	80	0	70
0	70	70	90	70	80	0	70	0	70
70	90	70	80	0	70	0	70	70	90
70	0	0	70	0	90	70	90	70	80
0	70	0	70	70	90	70	80	0	70
0	70	70	90	70	80	0	70	0	70
70	90	70	80	0	70	0	70	70	90
70	80	0	70	0	70	70	90	70	80
0	70	0	70	70	90	70	80	0	70
0	70	70	90	70	80	0	70	0	70
70									

Table S4 The theoretical "gene" sequence required to generate the illusion in Figure S9a (read from left to right, top to bottom; size: 1×141; unit: Hz).

0	70	0	70	70	90	70	80	0	70
0	70	70	90	70	80	0	70	0	70
70	90	70	80	0	70	0	70	70	90
70	80	0	70	0	70	70	90	70	80
0	70	0	70	70	90	70	80	0	70
0	70	70	90	70	80	0	70	0	70
70	90	70	80	0	70	0	70	70	90
70	80	0	70	0	70	70	90	70	80
0	70	0	70	70	90	70	80	0	70
0	70	70	90	70	80	0	70	0	70
70	90	70	80	0	70	0	70	70	90
70	80	0	70	0	70	70	90	70	80
0	70	0	70	70	90	70	80	0	70

0	70	70	90	70	80	0	70	0	70
70									

Table S5 The "gene" sequence generated from the illusion in Figure S9b (read from left to right, top to bottom; size: 1×141; unit: Hz).

40	50	40	50	40	50	40	50	40	50
40	50	40	50	40	50	40	50	40	50
40	50	40	50	40	50	40	50	40	50
40	50	40	50	40	50	40	50	40	50
40	50	40	50	40	50	40	50	40	50
40	50	40	50	40	50	40	50	40	50
40	50	40	50	40	50	40	50	40	50
40	50	40	50	40	50	40	50	40	50
40	50	40	50	40	50	40	50	40	50
40	50	40	50	40	50	40	50	40	50
40	50	40	50	40	50	40	50	40	50
40	50	40	50	40	50	40	50	40	50
40	50	40	50	40	50	40	50	40	50
40	50	40	50	40	50	40	50	40	50
40									

Table S6 The theoretical "gene" sequence required to generate the illusion in Figure S9b (read from left to right, top to bottom; size: 1×141; unit: Hz).

40	50	40	50	40	50	40	50	40	50
40	50	40	50	40	50	40	50	40	50
40	50	40	50	40	50	40	50	40	50
40	50	40	50	40	50	40	50	40	50
40	50	40	50	40	50	40	50	40	50
40	50	40	50	40	50	40	50	40	50
40	50	40	50	40	50	40	50	40	50
40	50	40	50	40	50	40	50	40	50
40	50	40	50	40	50	40	50	40	50
40	50	40	50	40	50	40	50	40	50
40	50	40	50	40	50	40	50	40	50
40	50	40	50	40	50	40	50	40	50
40	50	40	50	40	50	40	50	40	50

40	50	40	50	40	50	40	50	40	50
40									

Table S7 The "gene" sequence generated from the illusion in Figure S9c (read from left to right, top to bottom; size: 1×141; unit: Hz).

90	30	30	80	70	90	30	30	80	70
90	30	30	80	70	90	30	30	80	70
90	30	30	80	70	90	30	30	80	70
90	30	30	80	70	90	30	30	80	70
90	30	30	80	70	90	30	30	80	70
90	30	30	80	70	90	30	30	80	70
90	30	30	80	70	90	30	30	80	70
90	30	30	80	70	90	30	30	80	70
90	30	30	80	70	90	30	30	80	70
90	30	30	80	70	90	30	30	80	70
90	30	30	80	70	90	30	30	80	70
90	30	30	80	70	90	30	30	80	70
90	30	30	80	70	90	30	30	80	70
90	30	30	80	70	90	30	30	80	70
90									

Table S8 The theoretical "gene" sequence required to generate the illusion in Figure S9c (read from left to right, top to bottom; size: 1×141; unit: Hz).

90	30	30	70	80	90	30	30	80	70
90	30	30	80	70	90	30	30	80	70
90	30	30	80	70	90	30	30	80	70
90	30	30	80	70	90	30	30	80	70
90	30	30	80	70	90	30	30	80	70
90	30	30	80	70	90	30	30	80	70
90	30	30	80	70	90	30	30	80	70
90	30	30	80	70	90	30	30	80	70
90	30	30	80	70	90	30	30	80	70
90	30	30	80	70	90	30	30	80	70
90	30	30	80	70	90	30	30	80	70
90	30	30	80	70	90	30	30	80	70
90	30	30	80	70	90	30	30	80	70

90	30	30	80	70	90	30	30	80	70
90									

C. Validation of illusion generation accuracy

Based on our theoretical model (see Supplementary Note 5 for derivations), the radar system configuration used in this study involves 141 frequency-swept pulses. This corresponds to 141 opportunities for intra-pulse modulation of the metasurface. As established in the preceding section on frequency prediction, minor inaccuracies can occur in the generation of individual "genes" within the 141-element sequence under certain conditions.

However, we hypothesize that these isolated errors in the "gene" sequence may have a negligible impact on the quality of the final generated illusion. To validate this, we conducted a comparative simulation. We took the ground truth modulation sequences and the corresponding model-predicted sequences from the tests above. Using these two sets of "gene sequences," we simulated the resulting SAR illusions via our theoretical model. This allows for a direct comparison between the illusion generated from a perfect, pre-set sequence and the illusion generated from the network's predicted sequence.

As a primary example, Figure S10a displays the SAR illusion generated using the ground truth "gene" sequence (from Table S4), while Figure S10b shows the illusion generated using the model-predicted sequence (from Table S3). A qualitative visual inspection suggests that the two resulting images are nearly identical. To quantify this similarity, we employ the Structural Similarity Index (SSIM), a well-established metric for assessing the perceptual difference between two images. The SSIM index evaluates similarity based on three distinct components: luminance, contrast, and structure.

Let the SAR illusion generated from the ground truth frequency sequence be denoted as I_F, and the illusion generated from the network's predicted sequence be I_G. The luminance, contrast, and structural comparison functions are defined as $l(I_F, I_G)$, $c(I_F, I_G)$ and $s(I_F, I_G)$, respectively. The overall SSIM index is a function of these three independent components:

$$S(I_F, I_G) = f\big(l(I_F, I_G), c(I_F, I_G), s(I_F, I_G)\big) \tag{S46}$$

The luminance component is defined as:

$$l(I_F, I_G) = \frac{2\mu_{I_F}\mu_{I_G} + C_1}{\mu_{I_F}^2 + \mu_{I_G}^2 + C_1} \tag{S47}$$

The contrast component is defined as:

$$c(I_F, I_G) = \frac{2\sigma_{I_F}\sigma_{I_G} + C_2}{\sigma_{I_F}^2 + \sigma_{I_G}^2 + C_2} \tag{S48}$$

And the structural component is defined as:

$$s(I_F, I_G) = \frac{\sigma_{I_F I_G} + C_3}{\sigma_{I_F}\sigma_{I_G} + C_3} \tag{S49}$$

where μ_{I_F} and μ_{I_G} are the mean values of the two SAR images, and σ_{I_F} and σ_{I_G} are their standard deviations. $\sigma_{I_F I_G}$ represents the covariance of the two images. C_1, C_2, and C_3 are regularization constants used to prevent division by zero, typically set to small values such as 0.0001.

The SSIM index is then computed as:

$$\mathrm{SSIM}(I_F, I_G) = [l(I_F, I_G)]^{\alpha} \cdot [c(I_F, I_G)]^{\beta} \cdot [s(I_F, I_G)]^{\gamma} \tag{S50}$$

This algorithm is widely used, and functions for its direct computation are readily available in software packages such as MATLAB[S14].

Applying Equation S42 to our first test case, the SSIM between the SAR illusion from the ground truth sequence (Figure S10a) and the one from the model-predicted sequence (Figure S10b) is 99.99%. This result is remarkable; even with a "gene" sequence prediction accuracy of only 94.87%, the final illusion generation accuracy reaches 99.99%, indicating an exceptionally high degree of fidelity.

Similarly, for the results shown in Figure S10c and Figure S10d, the SSIM between the two images is 100%. For the final case shown in Figure S10e and Figure S10f, the SSIM is 99.61%, despite the "gene" generation accuracy being 98.82%. These results consistently demonstrate that even when the model's predicted control "genes" are not perfectly accurate, the resulting illusion conforms to the desired pattern with very high precision. From an application perspective, this robustness is highly advantageous, confirming that the model's performance fully meets the task requirements.

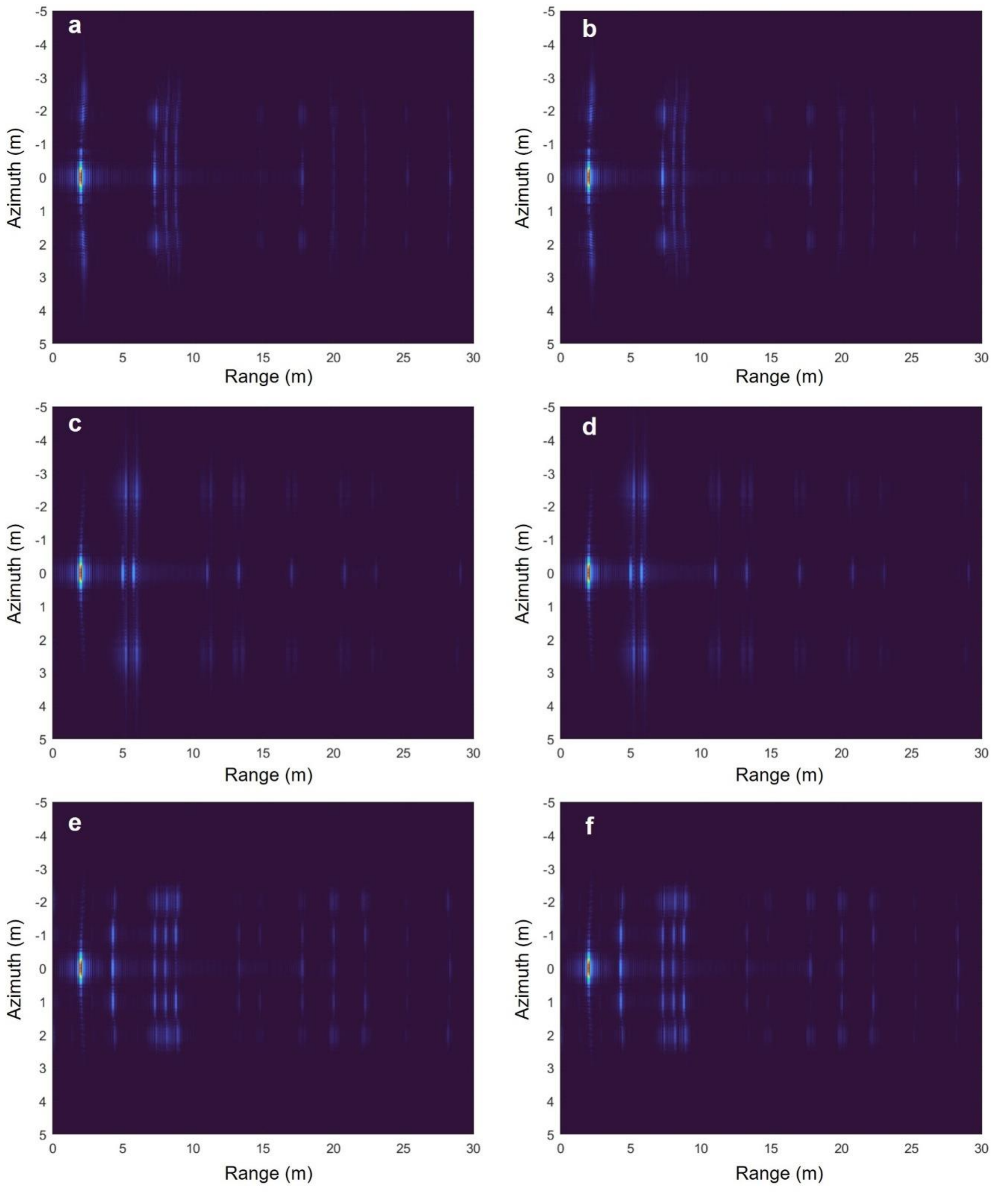

Figure S10 Comparison of SAR illusions generated from ground-truth and model-predicted gene sequences. Panels **a**, **c**, and **e** display the SAR illusions generated using the ground-truth frequency gene sequences for three distinct tasks. Panels **b**, **d**, and **f** show the corresponding illusions generated using the sequences predicted by the trained model. Each pair— (a, b), (c, d), and (e, f)—represents a direct comparison for an independent generation task. The visual results confirm that the model-predicted gene sequences can reproduce the target illusions with high fidelity, demonstrating the accuracy and effectiveness of the generation model.

Supplementary Note S9: Architecture and imaging performance of the rail SAR system

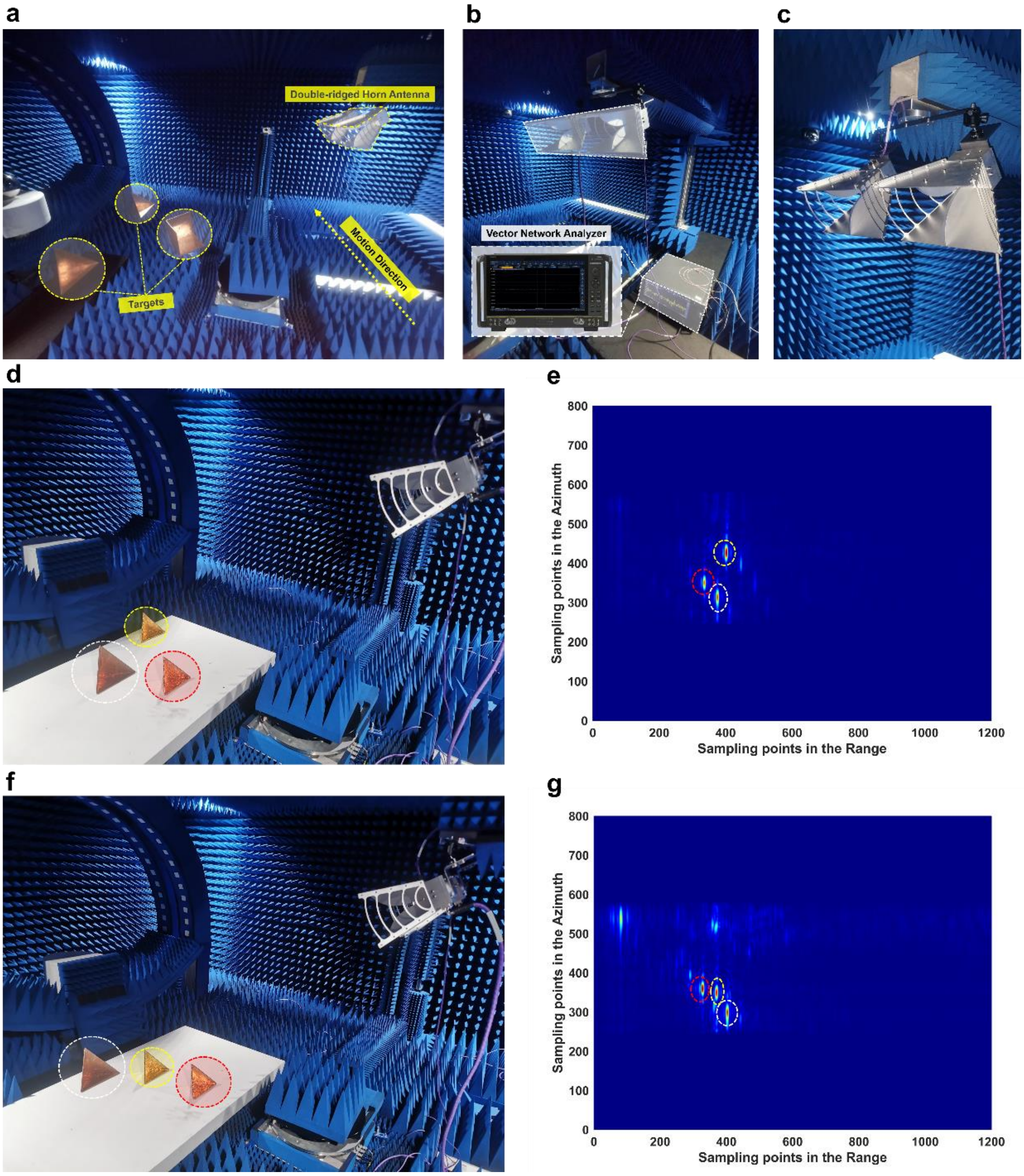

Figure S11. The laboratory-based SAR system: experimental setup and imaging results. **a,** Overall view of the experimental setup in the anechoic chamber. **b,** Key hardware components: the Vector Network Analyzer (VNA), transmitting/receiving (Tx/Rx) horn antennas, and a 3D motorized gantry. **c,** The two double-ridged horn antennas used in the experiment. **d,** Experimental scene with three corner reflectors in a triangular arrangement. **e,** Resulting SAR image for the triangular arrangement. **f,** Experimental scene with three corner reflectors in a linear arrangement. **g.** Resulting SAR image for the linear arrangement.

To validate the correctness of the theoretical derivations, a laboratory-based Synthetic Aperture Radar (SAR)

system is constructed within a microwave anechoic chamber to perform SAR imaging of targets within the scene. As illustrated in Figure S11a, this gantry-based SAR system consists of a three-dimensional (3D) stepper-motor-driven rail, a vector network analyzer (VNA), and a double-ridged horn antenna. This configuration enables the system to acquire data sequentially at each rail position in a "stop-and-go" mode. As shown in Figure S11b and Figure S11c, the specific model of the vector network analyzer is the Ceyear 3674 series. The double-ridged horn antenna operates over a frequency range of 1 to 18 GHz and has a beamwidth of 25 degrees. The 3D rail system's movement, including its speed and step size, is controlled via software.

In the experiment, the vector network analyzer is configured with a frequency sweep range from 5.5 GHz to 7.5 GHz, corresponding to a center frequency of $f_c = 6.5$ GHz and a bandwidth of $B = 2$ GHz. The sweep time for each measurement is set to $T_P = 1.002$ ms. The linear rail is programmed via its accompanying software to move over a total synthetic aperture length of $L = 1.4$ m with a step size of $\Delta l = 0.01$ m. This results in a total of $N_L = 141$ data acquisition points along the aperture.

As depicted in Figure S11d, a first SAR imaging experiment is conducted to validate the system. In this experiment, three corner reflectors are placed in the detection scene in a triangular arrangement. The base length of these corner reflectors is approximately 15 cm (which is noted to be smaller than the dimensions of the metasurface, 40 cm x 40 cm, used in later experiments). Using the 2D echo data acquired by the system and the implemented imaging algorithm, the final SAR image is generated, as shown in Figure S11e. In this image, the three targets are clearly visible and arranged in the expected triangular pattern, with well-defined contours and positions.

As shown in Figure S11f, a second SAR imaging experiment is then performed. For this test, the arrangement of the three corner reflectors is changed to a linear configuration. After processing the data with the same acquisition and imaging methods, the final imaging result is obtained, as shown in Figure S11g. The image clearly shows the three targets arranged in a line, again with sharp contours and accurate positions.

The successful outcomes of these two experiments serve to demonstrate the stability and accuracy of the experimental system. They confirm that the data acquisition method is effective and that the Range-Doppler imaging algorithm is correctly implemented. This provides a reliable hardware and software foundation for the subsequent measurement and validation of illusionary replica generation using the time-modulated metasurface.

Supplementary Note S10: Validation of similarity between simulation and experimental results

To validate the accuracy and reliability of the theoretical model proposed in this paper, we designed a series of comparative experiments aimed at quantitatively assessing the consistency between simulation predictions and real experimental data.

In each comparative experiment, we ensured the consistency of two key conditions: first, the radar parameters in the experimental environment (an anechoic chamber) perfectly matched the simulation settings; second, the "gene" sequence used to control the metasurface remained strictly identical in the simulation and the corresponding experiment.

For the quantitative assessment, we employed the same Structural Similarity Index (SSIM) metric used in Supplementary Note 8 to measure the similarity between the illusion images generated by the simulations and the experiments.

The results are presented in Figure S12. Panels a, c, and e display the SAR illusion images generated via theoretical simulation for three different "gene" sequences. Panels b, d, and f show the corresponding experimental results measured in an anechoic chamber under the control of the identical "gene" sequences. The calculated SSIM values for each pair are as follows:

1. The SSIM value between Figure S12a (simulation) and Figure S12b (experiment) is 94.35%.
2. The SSIM value between Figure S12c (simulation) and Figure S12d (experiment) is 93.87%.
3. The SSIM value between Figure S12e (simulation) and Figure S12f (experiment) is 94.84%.

The SSIM values for all comparison groups exceed 93%, indicating a high degree of consistency between the simulation results and the experimental data. This provides strong mutual validation for both the predictive capability of our theoretical model and the effectiveness of the physical experiments, thereby confirming the overall reliability of our proposed method.

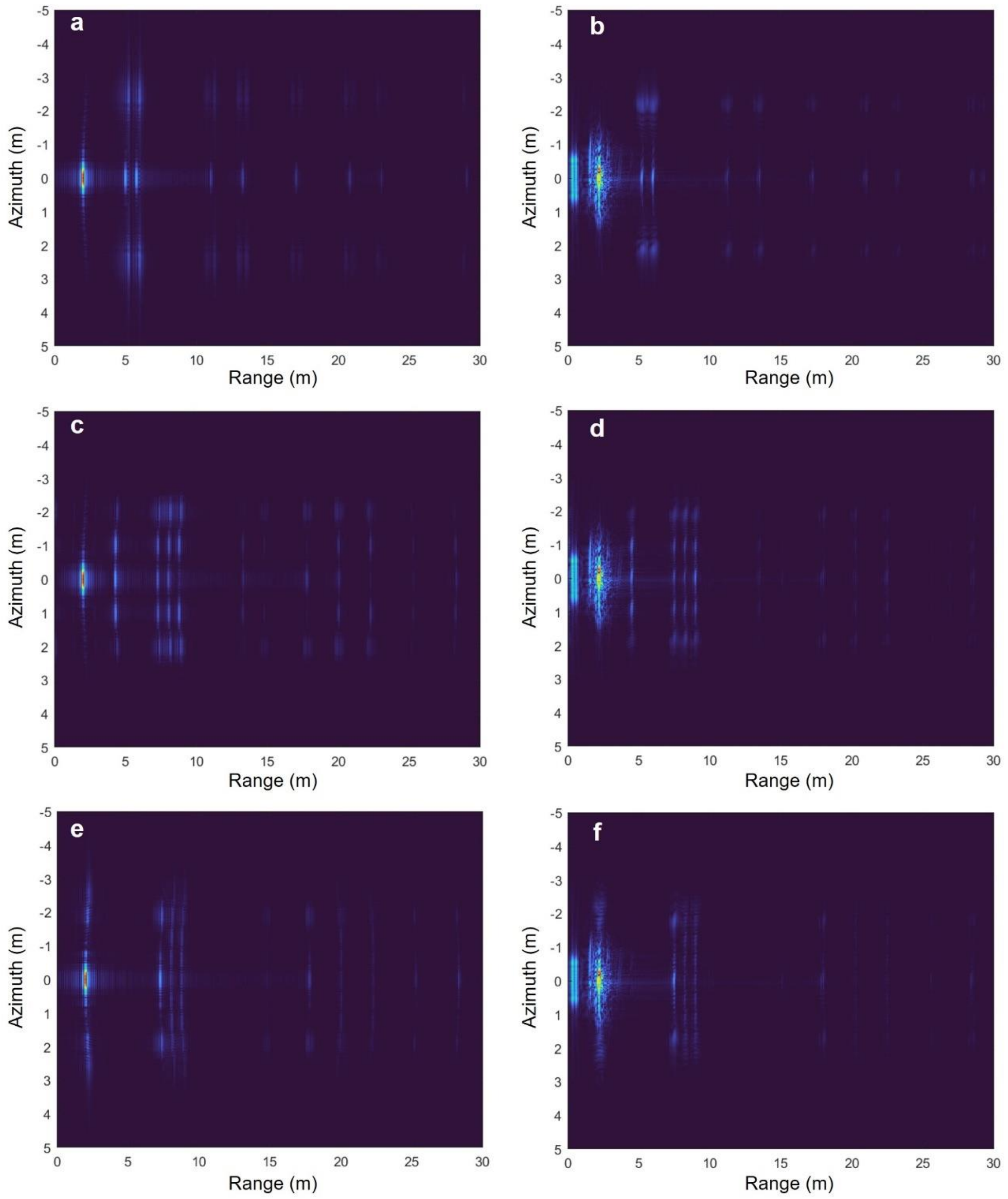

Figure S12 Comparison and validation of simulation and experimental results. Panels **a**, **c**, and **e** show the theoretical simulation results for three different modulation gene sequences. Panels **b**, **d**, and **f** display the corresponding results from physical experiments conducted using the identical gene sequences. Each pair— (a, b), (c, d), and (e, f)—forms an independent group for comparative validation, demonstrating the consistency between theory and practice.

Supplementary Note S11: Expansion of temporal modulation waveforms based on 1 bit amplitude coding metasurfaces

Although the 1-bit amplitude coding metasurface is fundamentally limited to switching between two discrete reflection states, the diversity of selectable temporal modulation waveforms can be significantly enhanced. By precisely manipulating the duty cycle and frequency of the square wave, or by synthesizing complex modulation sequences in accordance with Equation (1), a highly enriched library of candidate waveforms is generated.

To systematically demonstrate the expansion capabilities of the temporal modulation waveform library provided by this 1-bit architecture, we investigate square wave modulation signals across varying duty cycles. **Figure S13**a, c, and e depict the square wave modulation waveforms alongside their corresponding frequency spectra for duty cycles of 0.5, 0.7, and 0.3, respectively. The resulting modulation effects on a single radar pulse are presented in **Figure S13**b, d, and f. These analytical results clearly indicate that distinct temporal modulation waveforms yield highly differentiated one-dimensional electromagnetic illusion.

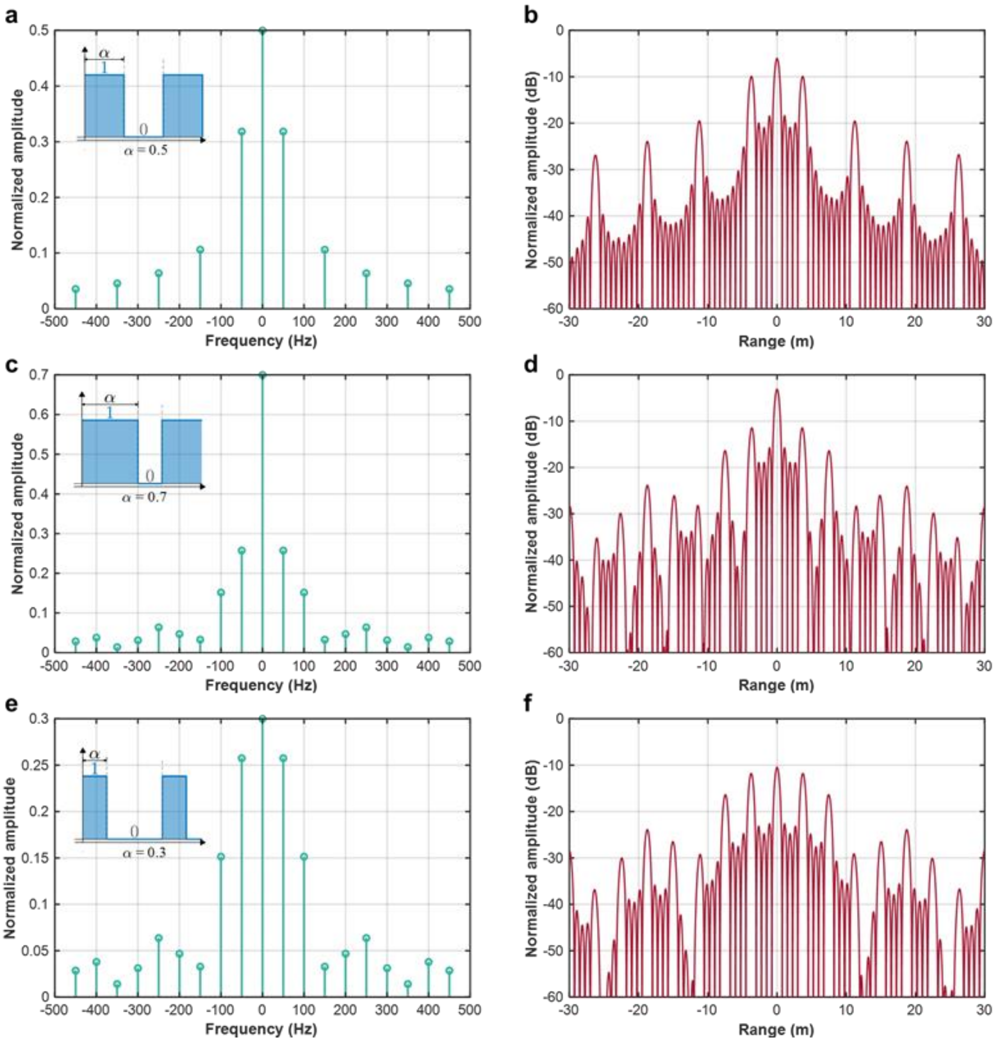

Figure S13 Expansion of temporal modulation waveforms based on 1 bit amplitude coding metasurfaces. a Frequency spectrum of the square wave temporal modulation waveform with a duty cycle of 0.5. b One dimensional range profile result after applying the modulation to a single pulse with a duty cycle of 0.5. c and d Frequency spectrum and the

corresponding one-dimensional range profile for a duty cycle of 0.7. e and f Frequency spectrum and the corresponding one-dimensional range profile for a duty cycle of 0.3.

Building upon this foundation, we further evaluate periodic sequence temporal modulation waveforms enabled by the 1-bit amplitude coding metasurface. Supplementary Figure S2a and d illustrate these waveforms with a defined sequence length of $M = 8$, while their respective frequency spectra are displayed in **Figure S14**b and e. When applied as independent gene units to modulate a single radar pulse, the corresponding one-dimensional range profiles post pulse compression are obtained, as shown in **Figure S14**c and f. These findings validate that, despite the physical constraint of possessing only two states, the 1-bit amplitude coding metasurface can execute complex and versatile waveform designs governed by Equation (1). Consequently, these configurations effectively function as specialized gene units to perform tailored and differentiated modulations on incident radar pulses.

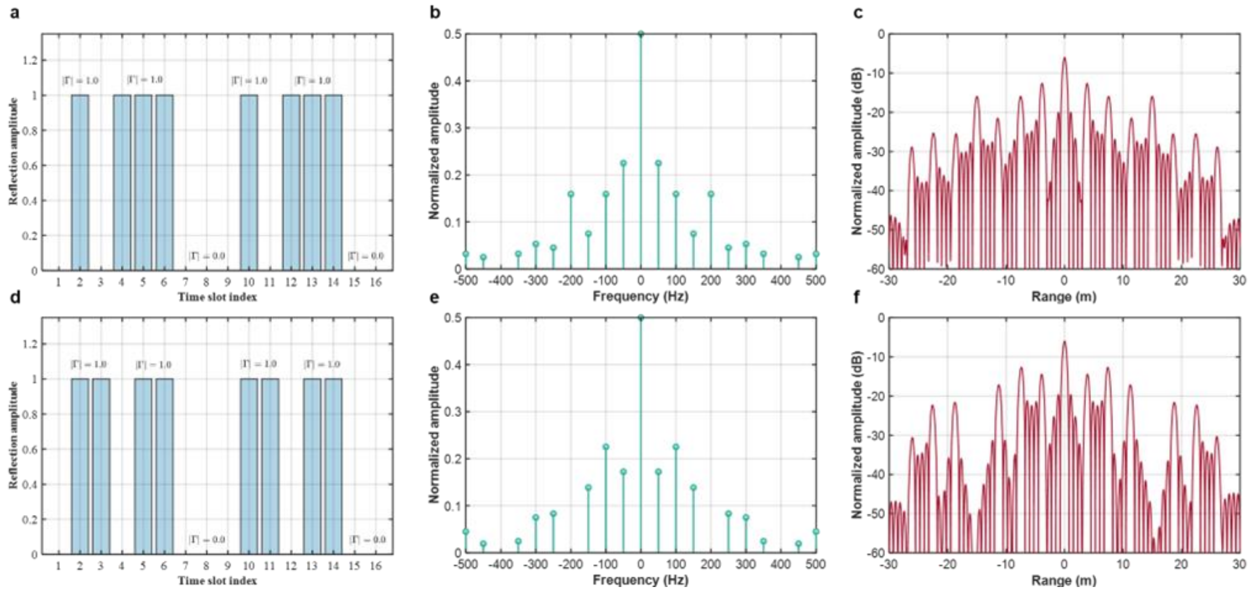

Figure S14 Expansion of temporal modulation waveforms based on 1 bit amplitude coding metasurfaces. a Periodic sequence temporal modulation waveform under coding scheme 1. b The corresponding frequency spectrum. c One dimensional range profile result after applying the modulation to a single pulse. d Periodic sequence temporal modulation waveform under coding scheme 2. e The corresponding frequency spectrum. f The corresponding one dimensional range profile result for a single modulated pulse.

Supplementary Note S12: Evaluation of illusion generation performance

To comprehensively evaluate the illusion generation mechanism and ensure the construction of highly realistic complex illusions with cross observation consistency in electronic countermeasure scenarios, relying solely on image centric macroscopic visual fidelity metrics (such as the SSIM) is insufficient. It is imperative to incorporate objective, physics-based data. Consequently, four distinct quantitative physics-based metrics are introduced to rigorously assess the generation performance: Relative Spatial Error ($E_{spatial}$), Carrier Suppression Ratio (CSR), Peak to Carrier Ratio (PCR), and Signal to Clutter Ratio (SCR).

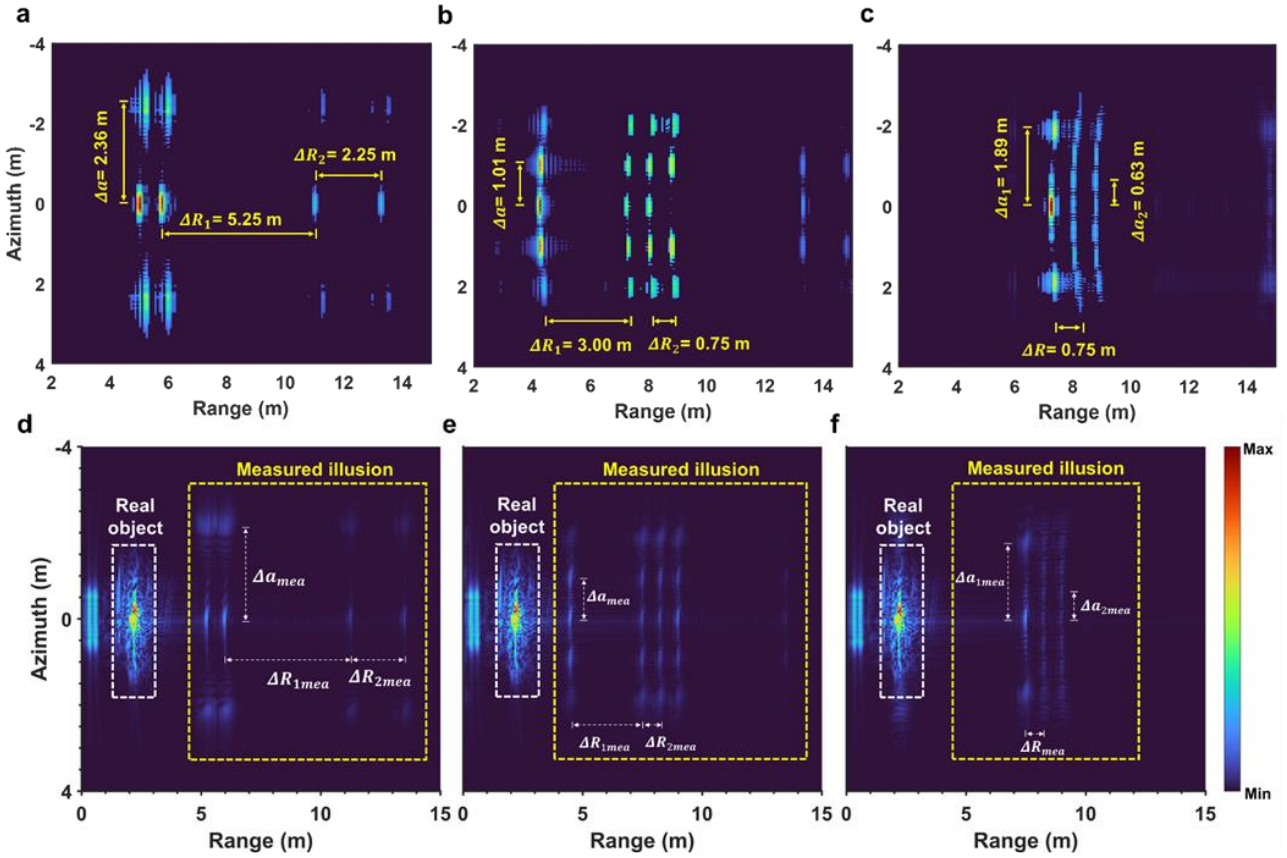

Figure S15 Predefined illusions and the corresponding experimental test results. a, b, and c The predefined illusion images for Task 1, Task 2, and Task 3, respectively. d, e, and f The corresponding experimental test results for Task 1, Task 2, and Task 3.

First, spatial localization accuracy is quantitatively assessed to verify whether the generated illusions appear exactly at the predefined coordinates. Specific distance features related to the false illusion distribution were extracted to compare the actual generated coordinates with the expected targets. For Task 1, comparing the expected distribution (Figure S15a) with the experimental measurement (Figure S15d), the measured range intervals are ΔR_{1mea} = 5.25 m and ΔR_{2mea} = 2.25 m, and the azimuth interval is Δa_{mea} = 2.26 m. This yields a relative spatial error $E_{spatial}$ of 4.23% compared to the expected target. Task 2 (Figure S15e), the measured intervals are ΔR_{1mea} = 3.075 m, ΔR_{2mea} = 0.75 m, and Δa_{mea} = 0.97 m, resulting in a relative error of 2.5% for the range direction generation and 3.96% for the azimuth direction generation. For Task 3 (Figure S15f), the measured intervals are

ΔR_{mea} = 0.75 m, Δa_{1mea} = 1.80 m, and Δa_{2mea} = 0.66 m, with a relative error of 4.76% for the azimuth direction. These results strictly remain within the 5% error threshold, explicitly demonstrating that the illusions are accurately synthesized at the designated locations.

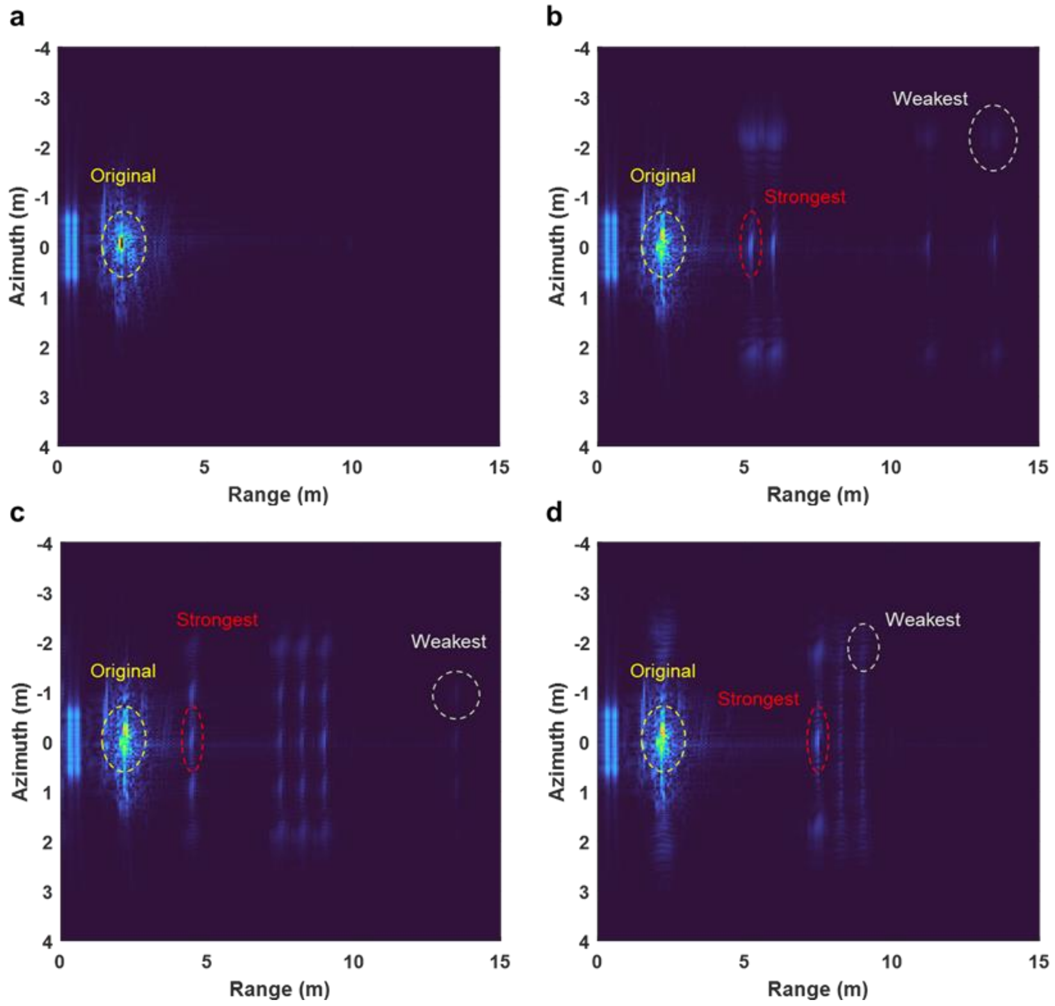

Figure S16 Experimental test results of illusion generation. a Synthetic aperture radar imaging without metasurface modulation. b, c, and d The corresponding experimental test results for Task 1, Task 2, and Task 3, respectively.

Second, the energy redistribution efficiency is evaluated. A fundamental physics-based metric is the amplitude variation of the original target before and after modulation, which proves that the metasurface successfully transfers energy from the original carrier to the false illusions. Due to the inherent fundamental wave leakage of the 1-bit square wave amplitude modulation, the original target will still exist in the synthetic aperture radar image but with significantly reduced energy. The Carrier Suppression Ratio is calculated by comparing the peak amplitude of the unmodulated target A_{unmod} with the modulated residual target A_{mod}. The formula is defined as:

$$\text{CSR (dB)} = 20\log_{10}\left(\frac{A_{mod}}{A_{unmod}}\right) \tag{S51}$$

The Carrier Suppression Ratios corresponding to Supplementary Figure S4b, c and d are -5.99 dB, -5.89 dB, and -5.57 dB, respectively. This indicates an average energy transfer efficiency of 73.81%, which excellently approaches the 75% theoretical maximum efficiency limit for 1 bit amplitude modulation.

Furthermore, the relative strength of the generated illusions is evaluated from an objective numerical perspective by calculating the Peak to Carrier Ratio. This metric compares the amplitude of the strongest illusion $A_{illusion}$ to the residual original carrier amplitude A_{mod} in each task. The calculation formula is:

$$\text{PCR (dB)} = 20\log_{10}\left(\frac{A_{illusion}}{A_{mod}}\right) \tag{S52}$$

Based on the results shown in Figure S16b, c and d, the Peak to Carrier Ratios for the strongest illusions are -11.1 dB, -12.2 dB, and -10.7 dB, respectively. Governed by the law of energy conservation, the total transferable energy is physically distributed among multiple predefined illusion positions depending on the specific task configuration.

Finally, to comprehensively evaluate the visibility of the illusions and eliminate subjective human visual biases, the Signal to Clutter Ratio is calculated. This metric assesses the ratio of the illusion intensity to the background noise intensity $A_{background}$, defined as:

$$\text{SCR (dB)} = 20\log_{10}\left(\frac{A_{illusion}}{A_{background}}\right) \tag{S53}$$

The Signal to Clutter Ratio was calculated for both the strongest and weakest illusions in each specific scenario. In **Figure S16**b, c, and d, the ratios for the strongest illusions against the background are 45.6 dB, 43.6 dB, and 45.8 dB, while the ratios for the weakest illusions are 27.8 dB, 32.5 dB, and 31.3 dB, respectively. These objective metrics further verify that the illusions generated by the metasurface possess excellent visibility and robustness against background noise in real experimental environments.

These complementary physics-based evaluation metrics are systematically summarized in Supplementary Table 9.

Table 9 Summary of quantitative physics-based evaluation metrics for illusion generation

Evaluation Task	Task 1	Task 2	Task 3
Max Relative Spatial Error (%)	4.23	3.96	4.76
Carrier Suppression Ratio (dB)	-5.99	-5.89	-5.57
Energy Transfer Efficiency (%)	74.85	74.28	72.30
Peak to Carrier Ratio (dB)	-11.1	-12.2	-10.7
Signal to Clutter Ratio (Strongest Illusion, dB)	45.6	43.6	45.8
Signal to Clutter Ratio (Weakest Illusion, dB)	27.8	32.5	31.3

Supplementary Note S13: Elucidation of the gene inspired modulation architecture and biomimetic mapping

To clarify the conceptual framework of the gene inspired architecture, it is essential to distinguish this approach from traditional evolutionary optimization algorithms. In this work, gene inspired refers to a biomimetic structural coding and recombination mechanism. This framework abstracts metasurface temporal modulation waveforms as fundamental gene units, which are spliced in a specific order to synthesize a complex two-dimensional modulation signal for radar illusion generation. The detailed physical foundation and mapping logic are articulated as follows.

1. Physical foundation: Two-dimensional signal processing

The proposed method specifically targets systems relying on temporal signal accumulation, with synthetic aperture radar serving as the primary representative. As the radar platform traverses an azimuth trajectory, it scans the target across N_L discrete spatial positions. At each position, a pulse is transmitted and the corresponding echo is stored. This scanning process establishes the slow time dimension, recording all transmitted and reflected signals throughout the synthetic aperture duration.

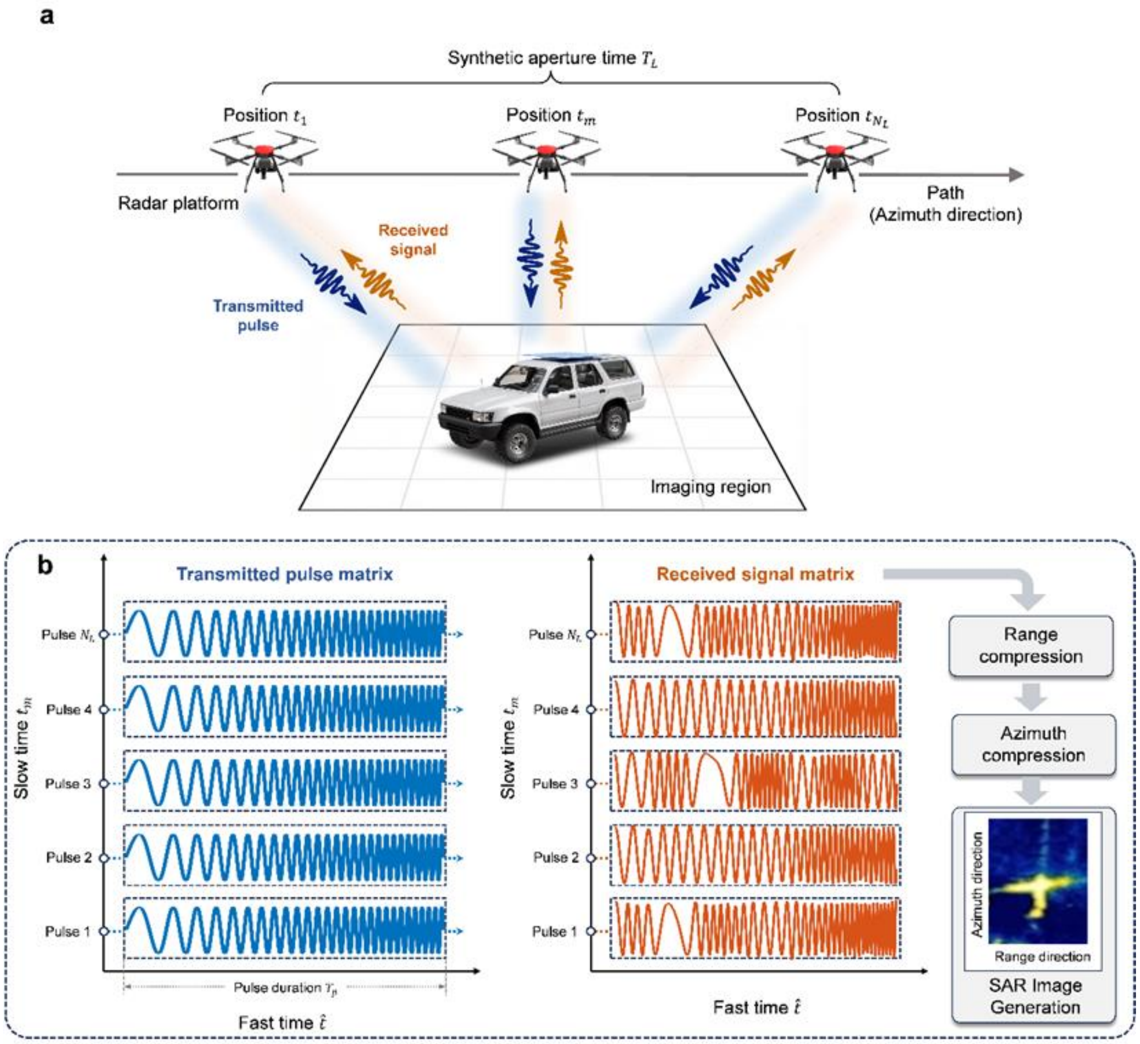

Figure S17 Schematic diagram of the synthetic aperture radar (SAR) imaging principle. a The ground target detection scenario of synthetic aperture radar. b Schematic of the transmitted and received signals and the imaging processing

workflow.

From a signal domain perspective, the radar acquires a total of N_L pulses, forming a two-dimensional data matrix. The vertical axis, or the slow time axis, corresponds to the pulse index during platform movement, determined by the radar velocity and pulse repetition interval. The horizontal axis, or the fast time axis, represents the rapid propagation of a single linear frequency modulated signal. The final imaging algorithm fundamentally relies on the coherent processing of this two-dimensional echo matrix. Consequently, the resulting image is not the product of a single pulse, but the result of the accumulation of all echo signals along the slow time trajectory.

2. The biomimetic mapping logic

By leveraging the inherent degrees of freedom within this two-dimensional signal structure, we establish a mapping between biological gene expression and the radar illusion generation mechanism.

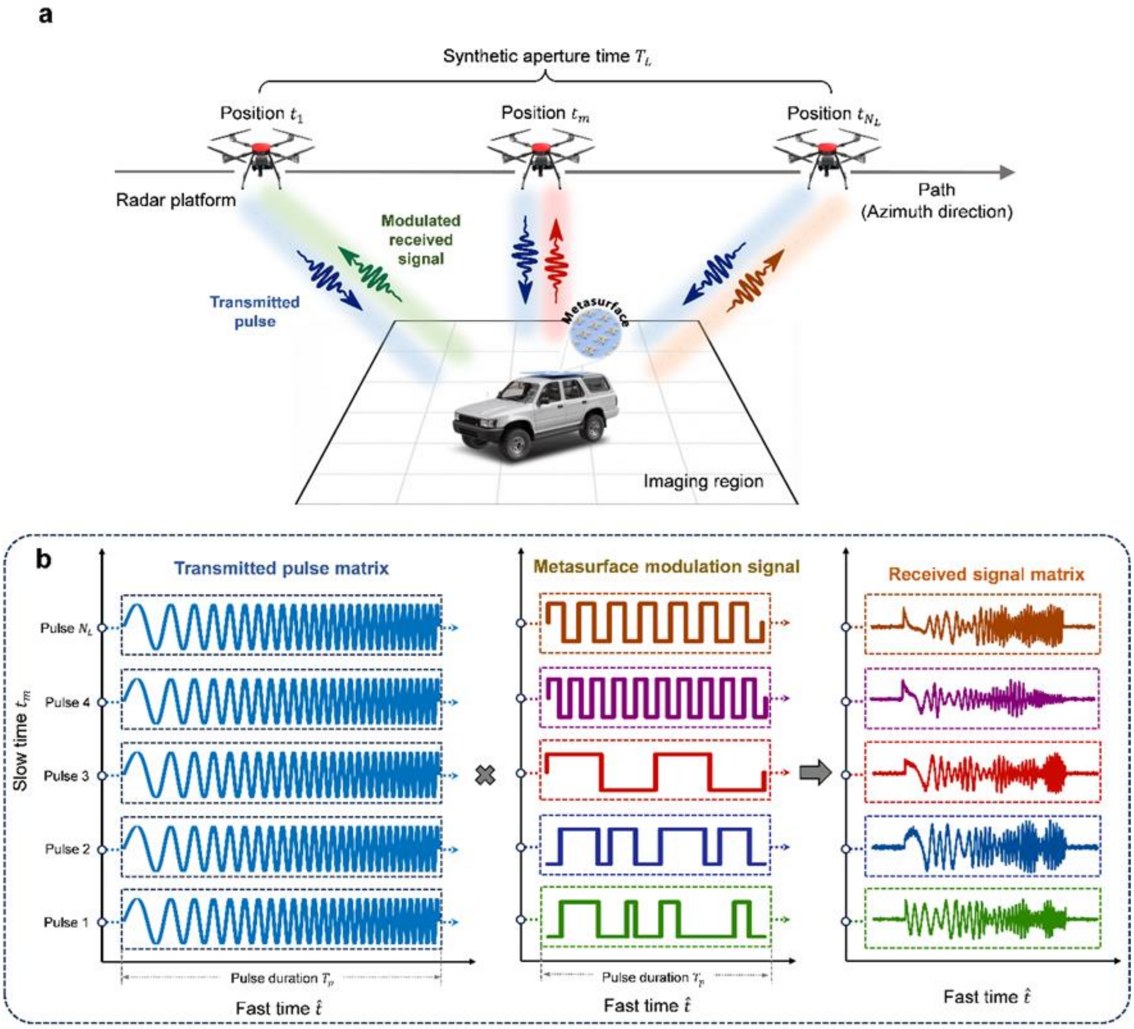

Figure S18 Schematic diagram of two-dimensional illusion generation via gene inspired metasurface temporal sequencing. a The two-dimensional illusion generation scenario utilizing gene inspired metasurface temporal sequencing. b Schematic of the transmitted signals, metasurface time modulation waveforms, and modulated echo signals.

- **Gene unit:** We define the specific metasurface temporal modulation waveform $\Gamma_n(\hat{t})$ applied within a single fast time pulse period (index n) as a basic gene unit. Analogous to a biological gene carrying

specific hereditary information, this metasurface waveform dictates the spectral characteristics of that individual echo signal.

- **Gene sequence:** Building upon the combinatorial potential of the slow time dimension, we implement a differentiated modulation strategy across all pulses. Instead of a fixed modulation, a distinct gene unit or temporal modulation waveform is selected for each of the N_L pulses. The ordered arrangement of these waveforms along the slow time axis constitutes the gene sequence $G = \{\Gamma_1(\hat{t}), \Gamma_2(\hat{t}), \ldots, \Gamma_{N_L}(\hat{t})\}$.
- **Phenotype expression:** In biology, phenotypes are the explicit expression of underlying genotypes. Similarly, when the specific gene sequence is applied to the radar signals, the received echoes undergo differential modulation. As the imaging algorithm processes this two-dimensional matrix, specific electromagnetic illusions are synthesized. Different gene combinations produce distinct biological traits; likewise, unique arrangements of these modulation units generate diverse illusions, enabling the precise control of illusion position, shape, and quantity.

Supplementary Note S14: Supplementary explanation of the Methods section

Spectrum Manipulation by Time-Modulated Metasurfaces

According to the Supplementary Note 2, and without loss of generality, we assume that the variation of the reflection coefficient's amplitude is a periodic function with a period of T_m. This period is divided into *M* discrete time slots of equal duration. The reflection coefficient's amplitude within the *m*-th time slot is denoted as Γ_m. Consequently, this time-varying function can be mathematically represented as:

$$\Gamma(t) = \sum_{m=0}^{M-1} \Gamma_m g(t - m\tau), (0 \le t < T_m)$$

Here, $g(t)$ is a periodic rectangular pulse function with a pulse width of $\tau = \frac{T_m}{M}$. By substituting the series for $g(t)$, the Fourier series expansion of the time-varying reflection coefficient $\Gamma(t)$ is derived as:

$$\Gamma(t) = \sum_{k=-\infty}^{\infty} \left[\frac{1}{M}\operatorname{sinc}\left(\frac{k\pi}{M}\right)\exp\left(-j\frac{k\pi}{M}\right)\left(\sum_{m=0}^{M-1} \Gamma_m \exp\left(-jk\frac{2m\pi}{M}\right)\right)\right]\exp(jk2\pi f_m t)$$

where $f_m = \frac{1}{T_m}$ is defined as the modulation frequency. According to the convolution theorem, multiplication in the time domain is equivalent to convolution in the frequency domain. Therefore, the spectrum of the reflected wave is given by the convolution of the incident spectrum and the reflection coefficient's spectrum:

$$E_r(f) = E_i(f) * \Gamma(f)$$

Mathematical model for the Genomic Combinatorial Time-modulation

Without loss of generality, we assume the inter-pulse modulation signal follows a periodic pattern with a period of T_a. This period is divided into *N* time slots, each with a width of τ_a, where each slot contains one radar pulse repetition interval. The temporal modulation signal within each slot is denoted as $\Gamma_n(\hat{t})$. This results in a 2D time-varying function that can be expressed as:

$$\Gamma_{2D}(\hat{t}, t_m) = \sum_{n=0}^{N-1} \Gamma_n(\hat{t}) g(t_m - n\tau_a), (0 \le t_m < T_a)$$

where $\Gamma_n(\hat{t})$ is the intra-pulse modulation waveform associated with the *n*-th modulation unit. Because each $\Gamma_n(t)$ follows the single-pulse model above, the complete modulation sequence can be expressed as:

$$\Gamma_{2D}(\hat{t}, t_m) = \sum_{n=0}^{N-1} \left[\sum_{m=0}^{M-1} \Gamma_m g(\hat{t} - m\tau)\right] g(t_m - n\tau_a), (0 \le t_m < T_a)$$

We denote the inter-pulse modulation signal as $F(t_m)$ and expand it using its Fourier series:

$$F(t_m) = \sum_{q=-\infty}^{+\infty} S_q \exp(j2\pi q f_a t_m)$$

where q is the order of the Fourier series harmonic, S_q is the coefficient of the q-th order harmonic, and f_a is the frequency of the inter-pulse modulation signal. Therefore, considering the genomic combinatorial time-modulation, the modified baseband echo is:

$$r_b(\hat{t}, t_m) = r(\hat{t}, t_m) \cdot \Gamma(\hat{t}) \cdot F(t_m)$$

References

[S1] Cui, T. J., Qi, M. Q., Wan, X., Zhao, J., & Cheng, Q. Coding metamaterials, digital metamaterials and programmable metamaterials. *Light Sci. Appl.* **3**, e218 (2014).

[S2] Zhang, L., Chen, X. Q., Liu, S. et al. Space-time-coding digital metasurfaces. *Nat. Commun.* **9**, 4334 (2018).

[S3] Ramaccia, D., Sounas, D. L., Alu, A., Toscano, A., & Bilotti, F. Phase-induced frequency conversion and Doppler effect with time-modulated metasurfaces. *IEEE Trans. Antennas Propag.* **68**, 1607-1617 (2019).

[S4] Zhang, X. G., Sun, Y. L., Yu, Q., Cheng, Q., Jiang, W. X., Qiu, C. W., & Cui, T. J. Smart Doppler cloak operating in broad band and full polarizations. *Adv. Mater.* **33**, 2007966 (2021).

[S5] Kozlov, V., Vovchuk, D., & Ginzburg, P. Broadband radar invisibility with time-dependent metasurfaces. *Sci. Rep.* **11**, 14187 (2021).

[S6] Meta, A., Hoogeboom, P., & Ligthart, L. P. Signal processing for FMCW SAR. *IEEE Trans. Geosci. Remote Sens.* **45**, 3519-3532 (2007).

[S7] Charvat, G. L., Kempell, L. C., & Coleman, C. A low-power high-sensitivity x-band rail sar imaging system [measurement's corner]. *IEEE Antennas Propag. Mag.* **50**, 108-115 (2008).

[S8] Feng, D., Tao, H., Yang, Y., & Liu, Z. Jamming de-chirping radar using interrupted-sampling repeater. *Sci. China Inf. Sci.* **54**, 2138-2146 (2011).

[S9] Feng, D., Xu, L., Pan, X., & Wang, X. Jamming wideband radar using interrupted-sampling repeater. *IEEE Trans. Aerosp. Electron. Syst.* **53**, 1341-1354 (2017).

[S10] Gu, J., Wang, Z., Kuen, J., Ma, L., Shahroudy, A., Shuai, B., ... & Chen, T. Recent advances in convolutional neural networks. *Pattern Recognit.* **77**, 354-377 (2018).

[S11] Dhillon, A., & Verma, G. K. Convolutional neural network: a review of models, methodologies and applications to object detection. *Progress in Artificial Intelligence* **9**, 85-112 (2020).

[S12] Hochreiter, S., & Schmidhuber, J. Long short-term memory. *Neural computation* **9**, 1735-1780 (1997).

[S13] Graves, A. Long short-term memory. Supervised sequence labelling with recurrent neural networks, 37-45 (2012).

[S14] MathWorks. Structural similarity (SSIM) index. https://www.mathworks.com/help/images/ref/ssim.html.
